# Impact of preanalytical variables on urinary cell-free DNA quality: Centrifugation, EDTA concentration, storage and thawing strategies

**DOI:** 10.1371/journal.pone.0356722

**Published:** 2026-08-27

**Authors:** Wenjing Li, Shan Guo, Zongning Zhou, Hongwei Peng, Yan Fu, Cong Zou, Xin Zhang, Shenjuan Li, Zhangyan Zhou, Kaiyu Qian, Shanshan Zhang

**Affiliations:** 1 Department of Biological Repositories, Zhongnan Hospital of Wuhan University, Wuhan, Hubei, China; 2 Human Genetic Resources Preservation Centre of Hubei Province, Wuhan, Hubei, China; 3 Department of Urology, Xingshan People’s Hospital, Yichang, Hubei, China; 4 Hubei Key Laboratory of Urological Diseases, Zhongnan Hospital of Wuhan University, Wuhan, Hubei, China; 5 Human Genetic Resources Preservation Centre of Wuhan University, Wuhan, Hubei, China; China Academy of Chinese Medical Sciences Institute of Chinese Materia Medica, CHINA

## Abstract

Urinary cell-free DNA (ucfDNA) holds promise for liquid biopsies, yet standardized preprocessing protocols remain to be fully established. Using pooled urine from healthy volunteers, we compared the effects of EDTA concentration (0–40 mM), centrifugation (0–2 spins), storage temperature and duration (RT, −20°C, −80°C), and thawing conditions (4°C, RT, 37°C) on ucfDNA yield and purity (%cfDNA score). The optimal protocol was subsequently validated using patient urine. Untreated whole urine (WU) and urine supernatant (US) both exhibited substantial ucfDNA degradation within 1 day at RT. For EDTA-treated WU, %cfDNA scores declined markedly owing to high-molecular-weight DNA contamination, and 20–40 mM EDTA compromised yields. In contrast, 10 mM EDTA-treated US optimally preserved both ucfDNA yields and %cfDNA scores for 7 days at RT, whereas 40 mM EDTA impaired yields. Frozen US showed thawing-method-independent stability, whereas frozen WU thawed at 4°C lost >60% of ucfDNA yields. Post-thawing secondary centrifugation further reduced ucfDNA recovery. For long-term storage, 10 mM EDTA-treated US maintained baseline-equivalent ucfDNA quality at −80°C for 3 months, but ucfDNA degraded after 2 months at −20°C. Patient urine validation confirmed that 10 mM EDTA-treated US frozen at −20°C and thawed at 37°C preserved ucfDNA quality comparable to baseline. This study offers practical guidelines and standardized preprocessing protocols for optimizing ucfDNA preservation. For temporary storage of urine intended for ucfDNA preservation, adding EDTA to WU is not advised; instead, we recommend supplementing 10 mM EDTA to US and storing it at RT ≤ 7 days or at −20°C ≤ one month. Long-term storage requires temperatures below −80°C. Rapid thawing can yield good ucfDNA quality.

## Introduction

Cell-free DNA (cfDNA) extracted from biological fluids has emerged as a potential biomarker for clinical diagnostics, offering noninvasive insights into genetic alterations, tumor dynamics, and various pathological conditions. Owing to its truly noninvasive collection, reduced volume limit, potential for repeated sampling, exemption from professional operation and minimal protein contamination, urine has attracted considerable attention as a source of cfDNA and is becoming a preferable alternative to tissue biopsies [1–3]. Urinary cell-free DNA (ucfDNA) has been reported to be a feasible candidate for the early detection of tumor-derived genetic mutations in various solid tumors, such as colon, lung, genitourinary, pancreatic, glioma, and hematopoietic cancers [3–9]. Despite the increasing interest in ucfDNA, there is a scarcity of standardized protocols for urine preprocessing, resulting in inconsistencies across studies and thereby restricting the successful application of ucfDNA [10–14]. This issue may arise because: (1) numerous microorganisms in urine can easily cause sample deterioration; (2) high nuclease activity makes ucfDNA prone to degradation; and (3) ucfDNA is highly fragmented [2,15].

The addition of preservatives to urine samples has been widely studied as a strategy to stabilize ucfDNA and prevent its degradation. The protective effects of various preservatives on ucfDNA have been investigated. These include ethylenediaminetetraacetic acid (EDTA) (with or without pH adjustment), the “AlloU” buffer, and commercial stabilizers such as Streck Urine Preservative, Urine Conservation Medium, Urine Conservation Buffer, and Urinary Analyte Stabilizer [1,5,11,12,15–18]. They were added to either unprocessed whole urine (WU) or urine supernatant (US) after centrifugation. Among these preservatives, EDTA stands out as the most cost-effective and readily available additive. Nevertheless, the use of EDTA varies greatly. The majority of previous studies added EDTA to the WU. Ruppert et al. directly collected the WU into urine cups (100 mL) containing 2 mL of EDTA (0.5 M, pH 8) [5]. Markus et al. added 0.8 mL of 0.5 M EDTA to 40 mL WU samples within one hour of collection, centrifuged 10 mL aliquots at 1600 × g for 10 min, and stored them at −80°C [2]. After collection, Mouliere et al. immediately placed the WU samples on ice after collection and added 0.5 M EDTA within one hour [8]. The samples were then centrifuged twice at 4°C, and the resulting supernatants were aliquoted into 2 mL microtubes for storage at −80°C. Oreskovic et al. mixed WU samples with EDTA and Tris-HCl (pH 7.5) to achieve final concentrations of 25 mM and 10 mM, respectively, as soon as possible after collection [19]. The treated WU samples were stored in DNA LoBind tubes at −80°C. In contrast to the aforementioned treatments, Lee et al. added 10 mM EDTA to US following centrifugation [1]. Moreover, the final concentration of EDTA varied from 5 mM to 40 mM [1,5,15,16,19,20].

Notably, pretreated urine samples are typically frozen below −20°C until further downstream processing [1,2,5,8,19]. Thawing frozen urine before extracting ucfDNA is essential. The thawing procedure was not described in some studies [2,8,12]. Murugesan et al. thawed frozen whole urine (FWU) samples treated with three preservatives at room temperature (RT) [16]. Lee et al. thawed EDTA-treated frozen urine supernatant (FUS) at 4°C and centrifuged the thawed supernatant again at 3000 × g for 10 min to remove impurities [1]. Nel et al. thawed 3 mL protected FUS aliquots for one hour at RT [15]. Oreskovic et al. thawed 50–200 mL of protected FWU at 37°C and centrifuged it at 8,000 × g for 5 min [19]. Jordaens et al. thawed 18–20 mL of protected FWU in a 37°C water bath for approximately 15 min [18]. Hosoi H et al. thawed 10 mL FUS at 37°C and centrifuged it at 16,000 × g at 4°C [9]. However, whether different thawing conditions affect the quality of ucfDNA remains unclear. In addition, studies investigating the impact of frozen storage conditions on the quality of ucfDNA are scarce. A comparison of fresh and thawed FWU samples supplemented with three preservatives after 24 weeks of storage at −80°C revealed no difference in ucfDNA yield [16]. Lee et al. demonstrated that the yields of ucfDNA from FUS with 10 mM EDTA stored at −70°C for three months were greater than those from FUS stored at −20°C [1]. However, the loss of ucfDNA was greater than 80%.

To address these issues, the primary objective of this study was to establish a systematic comparison of preanalytical variables under controlled conditions using pooled healthy volunteer (HV) urine. We initially evaluated the effects of different EDTA concentrations on ucfDNA yield and purity (%cfDNA score). EDTA was added to both unprocessed WU and centrifuged US, which were then stored at RT for 1, 3, and 7 days to identify the optimal concentration. Subsequently, we investigated the effects of three thawing methods for FWU, FUS and FUS with postthawing secondary centrifugation (FUSS), as well as the impact of storage conditions, on the quality of ucfDNA. Finally, the optimized protocol was validated in six patient samples as a proof-of-concept feasibility assessment. This study provides valuable insights into the optimal handling, storage, and thawing conditions for urine samples intended for ucfDNA extraction.

## Materials and methods

### Sample collection and processing

This study was approved by the Medical Ethics Committee of Zhongnan Hospital of Wuhan University (Approval No. 2017038, 2017038−2). Sample collection was performed in accordance with the Helsinki Declaration. Written informed consent was obtained from all HVs and patients with urological tumors prior to urine sample collection from January 20 to September 17, 2025. All individuals in this study have provided written informed consent (as outlined in the PLOS consent form) to authorize the publication of their case details.

The second morning urine (midstream) samples were collected in sterile 250 mL polypropylene containers between 9:00 am and 11:50 am to ensure temporal consistency, with each individual contributing 50–250 mL. All the samples were processed immediately according to specific preprocessing protocols. In the exploratory experiment of the preservation protocols, urine samples from HVs were pooled and thoroughly mixed in a 1000 mL sterile container to minimize interindividual variability and ensure uniform initial conditions [15,18]. The pooled urine was then aliquoted into equal portions of approximately 15 mL in sterile 50 mL centrifuge tubes, with triplicates prepared for each experimental condition. To validate the optimal preservation protocol, urine samples from patients with urological tumors were directly aliquoted into the respective groups.

### Different concentrations of EDTA added to WU and US

**Fig 1:**
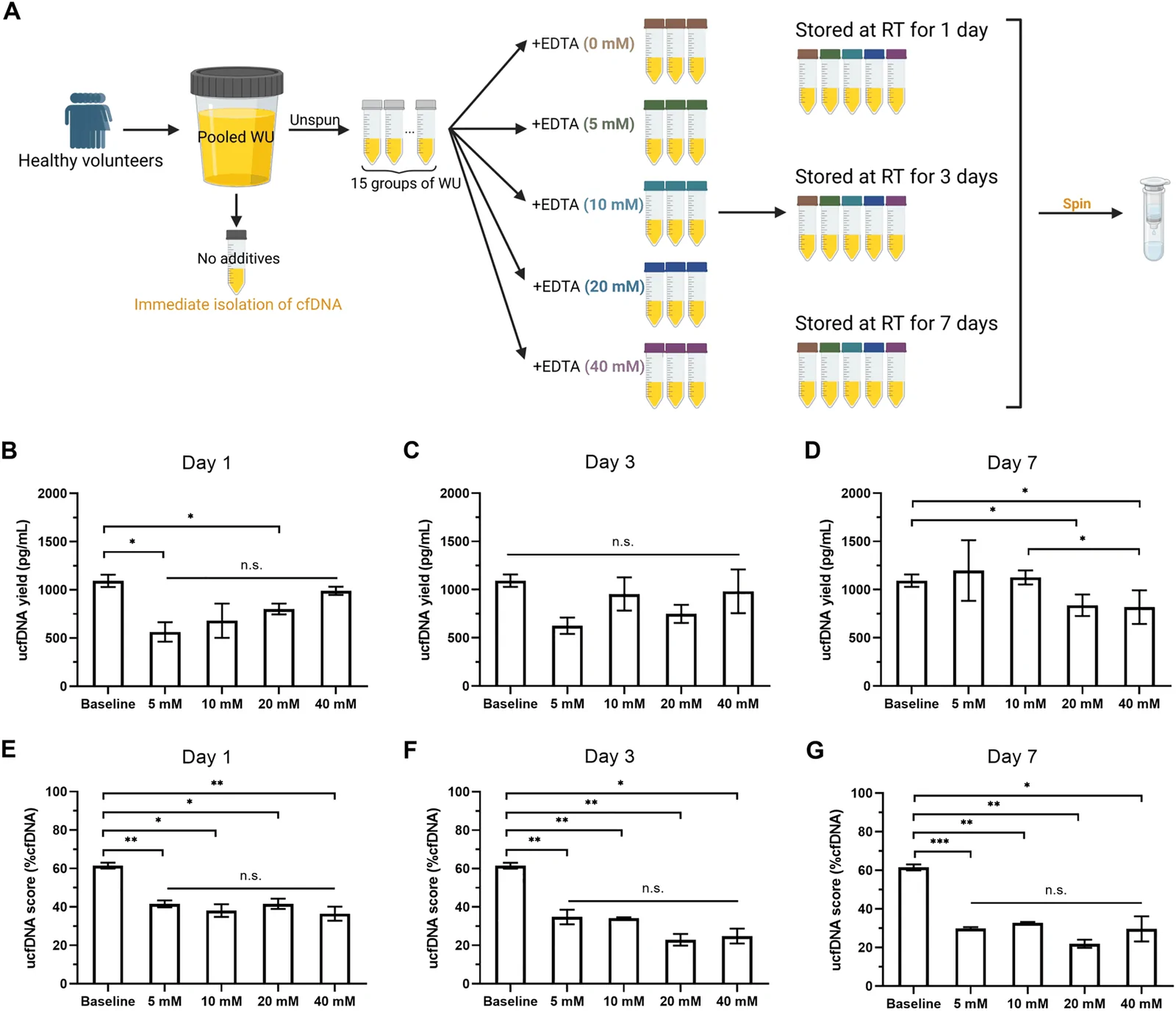
The protective effect of 0–40 mM EDTA on ucfDNA in WU samples stored at RT. (A) Schematic workflow of WU preprocessing, with triplicates conducted for each group. (B–D) The ucfDNA yields of EDTA-treated (5–40 mM) WU samples stored at RT for (B) 1 day, (C) 3 days, and (D) 7 days. (E–G) The %cfDNA scores of ucfDNA extracted from EDTA-treated (5–40 mM) WU samples stored at RT for (E) 1 day, (F) 3 days, and (G) 7 days. WU, whole urine; RT, room temperature; ucfDNA, urinary cell-free DNA; DNA yield, pg DNA/mL urine; n.s., nonsignificant; *p < 0.05. **p < 0.01. ***p < 0.001. Statistical significance was determined with repeated measures one-way ANOVA (B–C, E–G) and the Friedman test (D). Fig 1A was generated and permitted by BioRender (RRID:SCR_018361).

As shown in Fig 1A, WU samples collected from HVs were pooled and thoroughly homogenized. A total of 48 aliquots were distributed into 50 mL centrifuge tubes, with 3 aliquots designated as baseline controls for immediate ucfDNA isolation. The remaining 45 aliquots were randomly assigned to 15 groups and supplemented with 0.5 M EDTA (pH 8.0, Invitrogen, Cat# AM9260G) to achieve final concentrations of 0, 5, 10, 20, and 40 mM, followed by storage at RT for 1, 3, and 7 days prior to ucfDNA isolation. These storage durations were selected to simulate different sample delivery delay scenarios.

**Fig 2:**
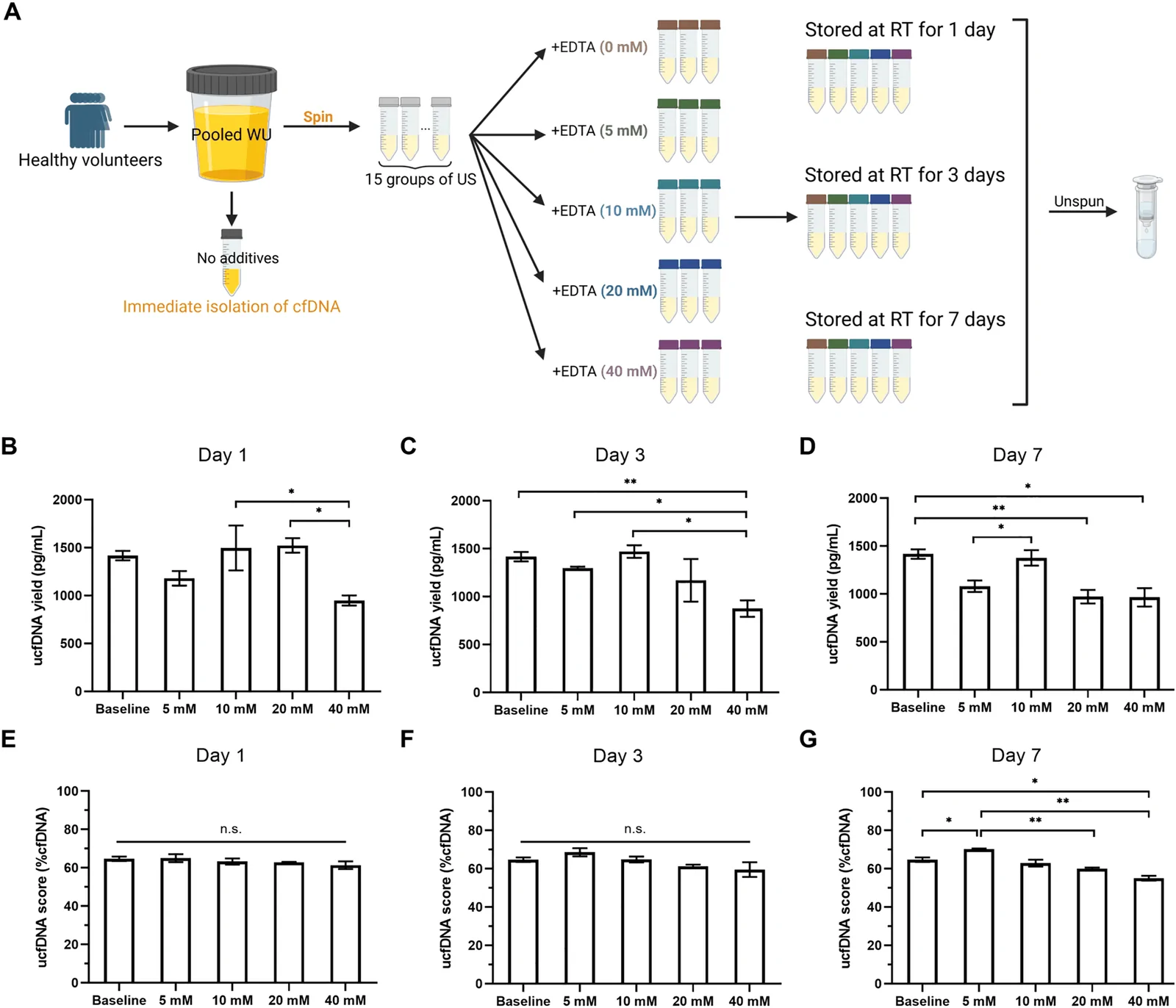
The protective effect of 0–40 mM EDTA on ucfDNA in US samples stored at RT. (A) Schematic workflow of US preprocessing, with triplicates conducted for each group. (B–D) The ucfDNA yields of EDTA-treated (5–40 mM) US samples stored at RT for (B) 1 day, (C) 3 days, and (D) 7 days. (E–G) The %cfDNA scores of ucfDNA extracted from EDTA-treated (5–40 mM) US samples stored at RT for (E) 1 day, (F) 3 days, and (G) 7 days. US, urine supernatant; RT, room temperature; ucfDNA, urinary cell-free DNA; DNA yield, pg DNA/mL urine; n.s., nonsignificant; *p < 0.05. **p < 0.01. Statistical significance was determined with repeated measures one-way ANOVA (C–D, E–G) and the Friedman test (B). Fig 2A was generated and permitted by BioRender.

Following the same protocol, WU samples collected from HVs were pooled and aliquoted as described above, with 3 aliquots reserved as baseline controls (Fig 2A). The remaining 45 aliquots were centrifuged at 3000 × g for 15 min at RT. The resulting US samples were carefully transferred into fresh 50 mL centrifuge tubes and processed via the same protocol as WU samples before ucfDNA isolation.

### Three thawing methods for frozen urine samples

**Fig 3:**
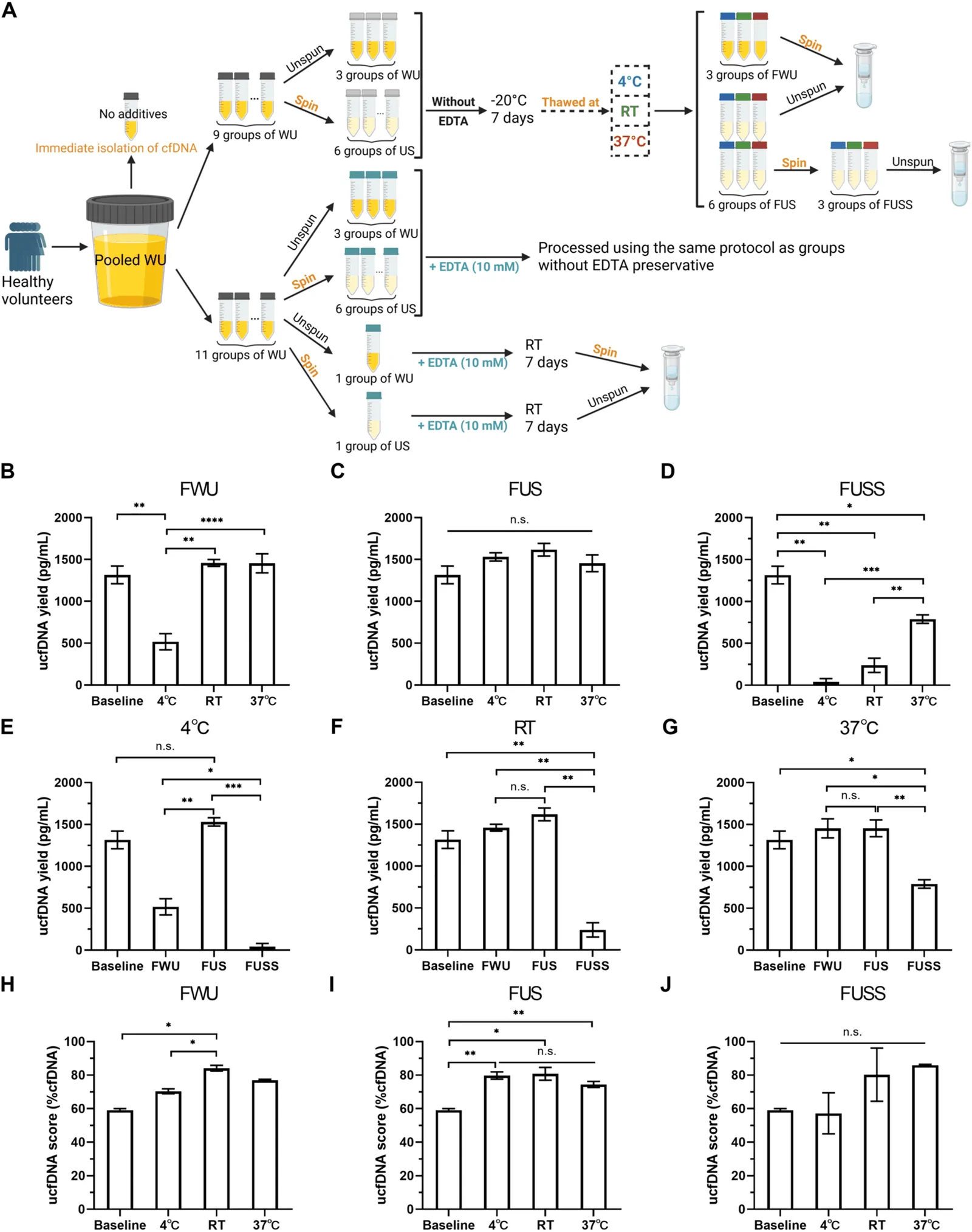
Evaluation of the ucfDNA quality of frozen urine under three thawing methods. (A) Schematic workflow of urine preprocessing, with triplicates conducted for each group. (B–D) In the absence of EDTA, the ucfDNA yields of the (B) FWU, (C) FUS and (D) FUSS samples stored at −20°C for 7 days were determined, followed by three thawing methods. (E–G) In the absence of EDTA, the ucfDNA yields of frozen urine samples stored at −20°C for 7 days followed by thawing at (E) 4°C, (F) RT and (G) 37°C. (H–J) The %cfDNA scores of ucfDNA extracted from the (H) FWU, (I) FUS and (J) FUSS samples stored at −20°C for 7 days followed by three thawing methods. ucfDNA, urinary cell-free DNA; DNA yield, pg DNA/mL urine; WU, whole urine; US, urine supernatant; FWU, frozen whole urine; FUS, frozen urine supernatant; FUSS, frozen urine supernatant with postthawing secondary centrifugation; RT, room temperature; n.s., nonsignificant; *p < 0.05. **p < 0.01. ***p < 0.001. ****p < 0.0001. Statistical significance was determined with repeated measures one-way ANOVA (B–G, I–J) and the Friedman test (H). Fig 3A was generated and permitted by BioRender.

WU samples collected from HVs were pooled and thoroughly homogenized. A total of 63 aliquots were distributed into 50 mL centrifuge tubes, with 3 aliquots designated as baseline controls for immediate ucfDNA isolation. The remaining 60 aliquots were randomly assigned to 20 groups, as shown in Fig 3A (n = 3 per group). For the 9 groups without EDTA preservative, the WU and US samples (3000 × g, 15 min, RT) were stored at −20°C for 7 days, followed by thawing in a 4°C refrigerator for 16 hours, an RT bench for 3.5 hours, or a 37°C water bath for approximately 15 min. This frozen condition (−20°C, 7 days) was chosen to mimic typical temporary storage after sample collection. After thawing, the FWU samples were centrifuged for ucfDNA extraction, whereas the FUS samples underwent either direct extraction or secondary centrifugation to eliminate impurities (FUSS). A total of 11 groups were treated with 10 mM EDTA. Two of these groups (one WU and one US) were stored at RT for 7 days before ucfDNA isolation. The remaining 9 groups were processed following the same procedures as those without EDTA.

### Different storage conditions

WU samples collected from HVs were pooled and thoroughly homogenized. A total of 21 aliquots were distributed into 50 mL centrifuge tubes, with 3 aliquots designated as baseline controls for immediate ucfDNA isolation. The remaining 18 aliquots were centrifuged at 3000 × g for 15 min at RT. The resulting US samples were carefully transferred into fresh 50 mL centrifuge tubes supplemented with 10 mM EDTA, followed by storage at −20°C or −80°C for one to three months (n = 3 per group, Fig 6A). The frozen US samples were thawed at 37°C for 15 min and subjected to direct ucfDNA extraction.

### The performance of the optimal preservation protocol in patient urine samples

WU samples were collected from six patients with urological tumors prior to surgical intervention. The clinical information of the patients is presented in Table 1. In addition to the baseline control group with immediate ucfDNA isolation, the optimal preservation protocol was validated using US samples supplemented with 10 mM EDTA, frozen at −20°C for 7 days, and then thawed at 37°C.

**Table 1:** The clinical information of patients.

Patient ID	Sex	Diagnosis	Stage
1	F	Renal pelvis cancer	pT3N0M0
2		M	Prostate cancer	pT2Nx
3			M	Primary hyperaldosteronism	N/A
4				M	Prostate cancer	pT3bN1
5					M	Bladder cancer	pT1N0M0
6						M	Bladder cancer	pT2N0M0

F, female; M, male; N/A, not available.

### ucfDNA extraction and evaluation

Several commercial kits are available for ucfDNA extraction [1,20]. Among these, the Quick-DNA™ Urine Kit (Zymo Research, Cat# D3061) was selected because of its capacity to process large sample volumes, with a maximum input of 40 mL of urine per standard preparation. ucfDNA extraction was performed following the manufacturer’s protocol. The procedure involved two main steps: (1) preconcentration of ucfDNA using magnetic beads and (2) subsequent purification through silica-based spin columns. The purified ucfDNA samples were eluted in 20–40 µL of elution buffer and either measured immediately or stored at −80°C for subsequent qualitative and quantitative analysis.

The total DNA (TDNA) quantification, separate cfDNA quantification and %cfDNA scores were determined via an Agilent 4200 TapeStation system (written:SCR_018435) with the Cell-free DNA Screen Tape assay (Agilent, Cat# 5067–5630). The DNA yield was calculated via the following formula: DNA yield (pg/mL urine) = C × V_1_/V_2_ (C, DNA concentration measured by Agilent 4200 [pg/µL]; V_1_, volume of elution buffer [μL]; V_2_, volume of urine [mL]). The %cfDNA score reports the percentage of cfDNA fragments (50–700 bp) in the total sample, providing a reference for quick determination of cfDNA sample quality [21]. A high %cfDNA score indicates a high‑quality sample with very little high‑molecular‑weight DNA (HMW DNA, > 800 bp) [21,22]. HMW DNA can be co‑extracted with cfDNA and can interfere with the sensitivity of detection techniques as well as downstream processes, such as next‑generation sequencing [11,23,24]. The electropherogram profiles and gel images of ucfDNA were generated via Agilent 4200 TapeStation software.

### Statistical analysis

All the statistical analyses were performed via GraphPad Prism (version 8.0.2; GraphPad Software Inc., RRID: SCR_002798). Given that pooled urine samples were aliquoted into equal portions and assigned to experimental groups, we selected the “each row represents matched data” option for the experimental design. Data normality was assessed via the Shapiro-Wilk test. In accordance with Prism’s recommendations, we chose not to assume sphericity. For multiple related group comparisons, we employed either repeated measures one-way ANOVA (RM one-way ANOVA) with Geisser-Greenhouse correction for normally distributed data or the Friedman test for nonparametric data distributions. In cases of significant overall results, post hoc pairwise comparisons were performed with appropriate correction: Tukey’s or Dunnett’s test for parametric RM one-way ANOVA, and Dunn’s test for nonparametric Friedman post hoc comparisons. For two related group comparisons, we employed a paired *t* test or Wilcoxon test. Statistical significance was defined as *p* < 0.05 for all tests. The figures were generated via GraphPad Prism. For all key comparisons, we calculated appropriate effect size measures using IBM SPSS Statistics 20, following established guidelines in the literatures [25,26]. We reported partial η² for RM one-way ANOVA, Kendall’s W for Friedman tests, Hedges’ g for paired t-tests, and r for Wilcoxon tests (see S1 File).

## Results

### Evaluation of ucfDNA Preservation in EDTA-treated WU

The preservation of ucfDNA was initially evaluated in WU samples treated with varying EDTA concentrations (0, 5, 10, 20, and 40 mM) after storage at RT for 1, 3, and 7 days. WU samples collected from HVs were pooled, thoroughly homogenized, and assigned to one baseline group and fifteen treatment groups (Fig 1A). Complete ucfDNA degradation occurred in the untreated (0 mM EDTA) WU samples after 1 day of RT storage (S1A Fig). Consequently, the DNA yields and %cfDNA scores for the 0 mM EDTA group were excluded from subsequent analyses.

All EDTA-treated groups (5–40 mM) maintained detectable ucfDNA throughout the 7-day storage period. On day 1, the 5 mM and 20 mM EDTA groups exhibited significant differences from the baseline (p = 0.0292 and p = 0.0293, respectively; Fig 1B). Nevertheless, no significant differences in ucfDNA yield were found among any EDTA-treated groups after 1 or 3 days of RT storage (Fig 1B and 1C). After 7 days of RT storage, the 20 mM and 40 mM groups yielded significantly lower ucfDNA than the baseline group (*p* = 0.0389 and *p* = 0.0201, respectively; Fig 1D). Additionally, the 40 mM group showed a notable decrease compared with the 10 mM group (p = 0.0269, Fig 1D). In particular, the %cfDNA scores decreased progressively in all EDTA-treated WU (61.51 ± 1.54 to 32.38 ± 7.04), which was correlated with ucfDNA reduction and TDNA accumulation (Figs 1E, 1G and S1C). Collectively, these results revealed a key limitation of EDTA in WU. Although EDTA preserved ucfDNA yield, it failed to prevent progressive HMW DNA contamination, as indicated by the declining %cfDNA scores.

### Evaluation of ucfDNA Preservation in EDTA-treated US

The preservation of ucfDNA was evaluated in US samples treated with varying EDTA concentrations (0, 5, 10, 20, and 40 mM) after storage at RT for 1, 3, and 7 days. WU samples collected from HVs were pooled, thoroughly homogenized, and assigned to one baseline group and fifteen treatment groups. After centrifugation, the resulting US samples were further processed (Fig 2A). Substantial ucfDNA degradation occurred in untreated (0 mM EDTA) US samples after 1 day of RT storage (S1B Fig).

Throughout the 7-day storage period, the ucfDNA yield in the 5 mM EDTA group was lower than that in the 10 mM EDTA group (1417 ± 49.33 pg/mL), and the ucfDNA yield in the 40 mM group was obviously lower than that in the other groups (Fig 2B–2D). The 5–20 mM EDTA groups maintained ucfDNA yields comparable to those at baseline, and no significant differences were observed after 1 day and 3 days of RT storage (Fig 2B and 2C). After 7 days of RT storage, the 20 mM and 40 mM groups presented lower ucfDNA yields than did the baseline group (Fig 2D). Throughout the 7‑day storage period, the 5 mM EDTA group maintained the highest %cfDNA scores. These scores were comparable to those of the 10 mM EDTA group and both were close to baseline (64.71 ± 1.20) (Fig 2E–2G). In all the EDTA-treated groups (5–40 mM), the %cfDNA scores were similar to those at baseline after 1 day and 3 days of RT storage (Fig 2E and 2F). However, the 40 mM EDTA group presented significantly lower %cfDNA scores (55.04 ± 1.27) than did the baseline group after 7 days of RT storage (*p* = 0.0127, Fig 2G). These findings indicated that 10 mM EDTA in US optimally balanced ucfDNA yield and purity, whereas 40 mM EDTA adversely affected both metrics.

### Evaluation of ucfDNA extracted from frozen urine samples via three thawing methods

We assessed the impact of three thawing methods on the quality of ucfDNA extracted from frozen urine samples, both with and without 10 mM EDTA. WU samples collected from HVs were pooled, thoroughly homogenized and assigned to one baseline group and twenty treatment groups (Fig 3A).

**Fig 4:**
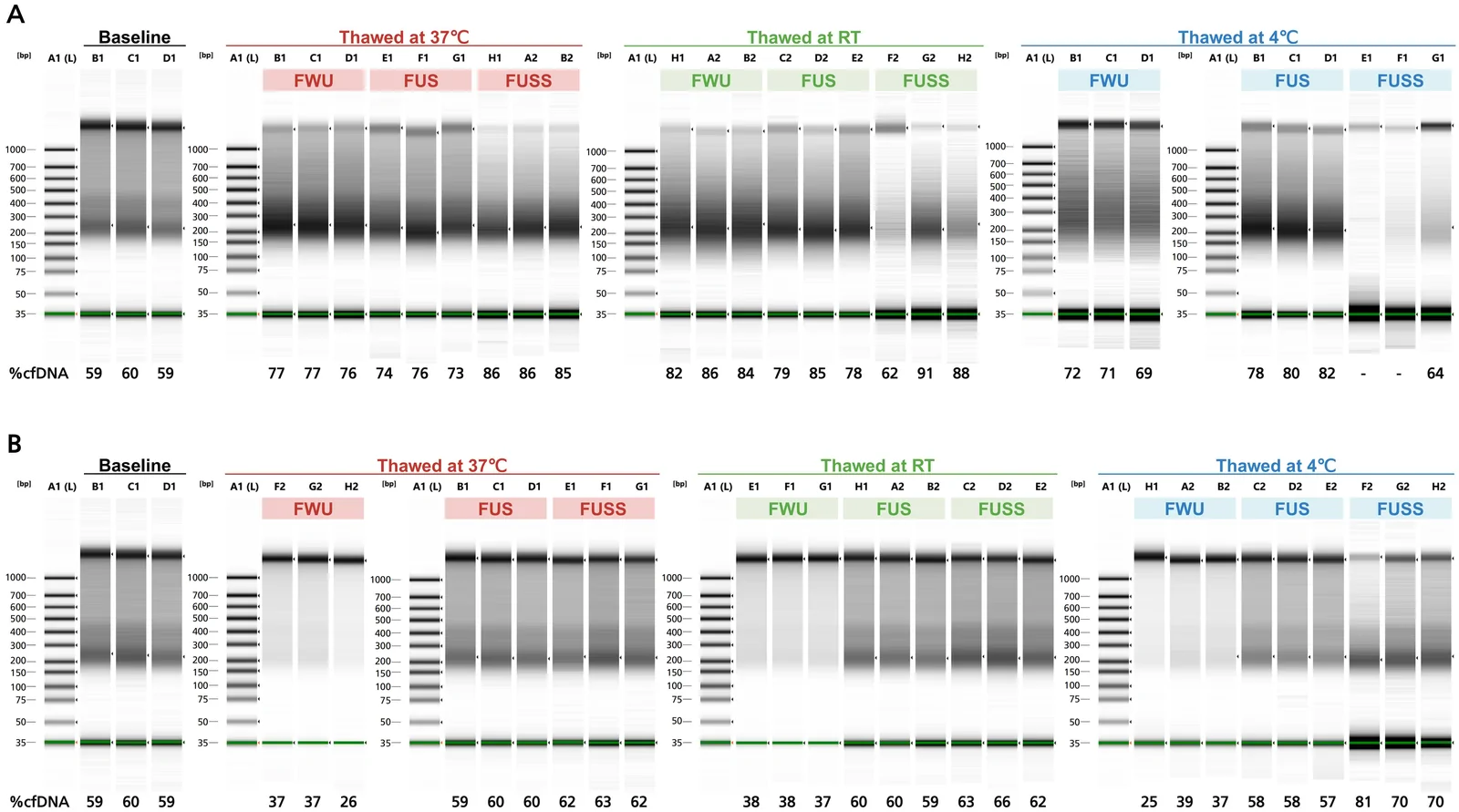
Gel images of ucfDNA extracted from frozen urine samples. (A) In the absence of EDTA, the gel images of ucfDNA extracted from the FWU, FUS and FUSS samples were stored at −20°C for 7 days, followed by three thawing methods. (B) In the presence of 10 mM EDTA, gel images of ucfDNA extracted from the FWU, FUS and FUSS samples stored at −20°C for 7 days followed by three thawing methods. To facilitate comparison, gel images of the baseline ucfDNA from the thawing experiments are included in both figures. The concentrations of the two FUSS samples without EDTA, thawed at 4°C, were outside the functional range for %cfDNA and the assay, displaying a “-” sign in the DNA band. Nevertheless, the %cfDNA scores for these two samples were provided in the region table. ucfDNA, urinary cell-free DNA; FWU, frozen whole urine; FUS, frozen urine supernatant; FUSS, frozen urine supernatant with postthawing secondary centrifugation.

In the absence of EDTA, the FUS samples presented higher ucfDNA yields postthawing than the baseline samples, with no significant differences among the three thawing methods (Fig 3C). The FWU samples thawed at 4°C presented a dramatic decrease in the ucfDNA yield, with a 60.70% loss (Fig 3B), and the FUSS samples yielded poorly across all methods (Fig 3D). When thawed at 4°C, only the FUS samples maintained ucfDNA yields comparable to those at baseline (Fig 3E). Upon thawing at RT and 37°C, the FWU and FUS samples performed similarly (Fig 3F and 3G). Despite the yields of ucfDNA being comparable to those of the baseline, the HMW DNA band became weaker, indicating a noticeable trend toward degradation in the gel images (Fig 4A). Compared with the FWU and FUS samples, the FUSS samples presented notably lower ucfDNA yields across the three thawing methods, and substantial degradation was shown in the gel images (Figs 3E, 3G and 4A). Nearly all the frozen urine samples without EDTA pretreatment presented higher %cfDNA scores than did the baseline samples after thawing (Fig 3H–3J). Compared with the baseline samples, the FUS samples presented obviously higher %cfDNA scores, with no significant differences observed among the three thawing methods (Fig 3I).

**Fig 5:**
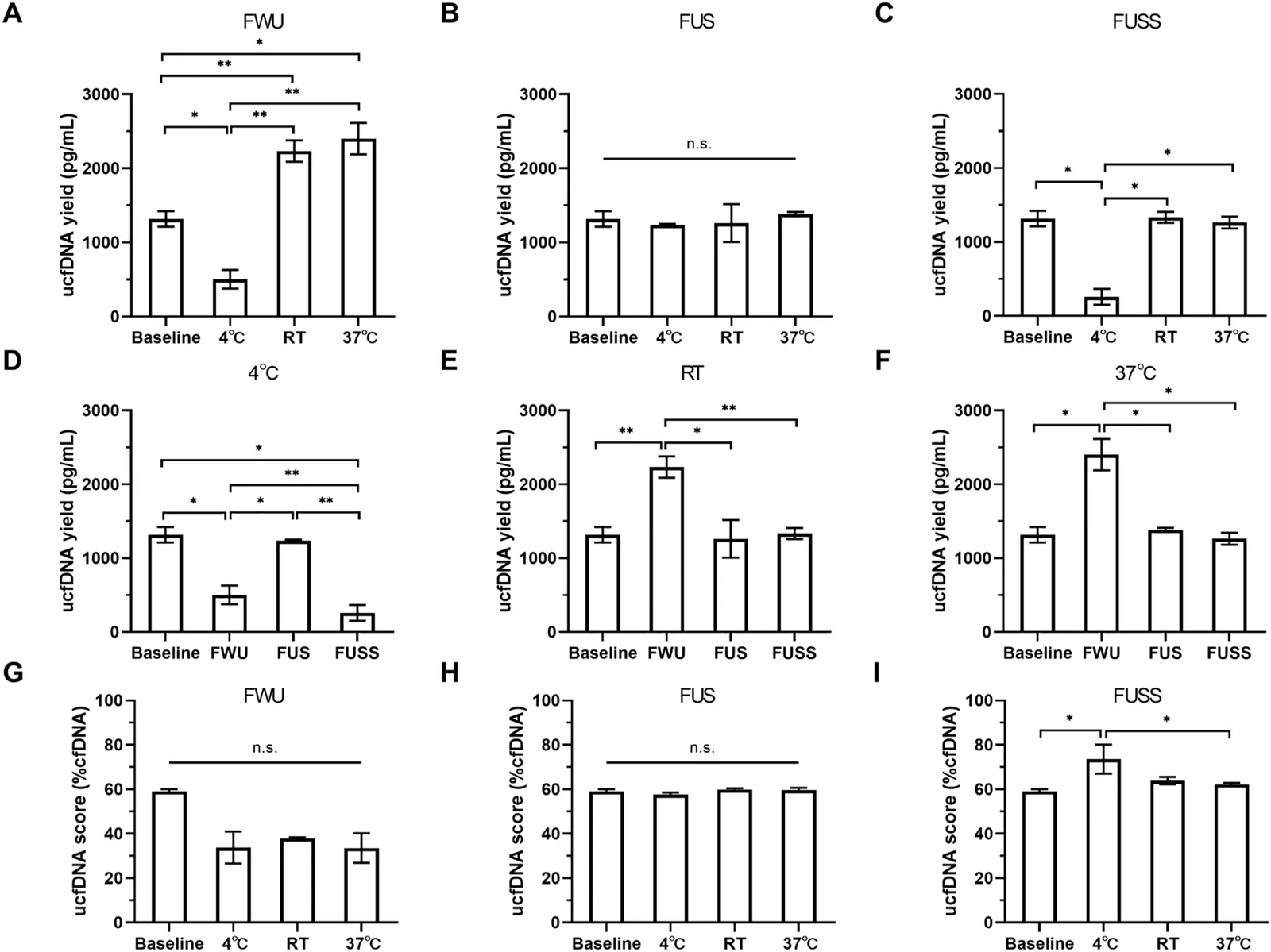
Evaluation of the ucfDNA quality of 10 mM EDTA-treated frozen urine under three thawing methods. (A–C) In the presence of EDTA, the ucfDNA yields of the (A) FWU, (B) FUS and (C) FUSS samples stored at −20°C for 7 days followed by three thawing methods. (D–F) In the presence of EDTA, the ucfDNA yields of frozen urine samples stored at −20°C for 7 days followed by thawing at (D) 4°C, (E) RT and (F) 37°C. (G–I) The %cfDNA scores of ucfDNA extracted from the (G) FWU, (H) FUS and (I) FUSS samples stored at −20°C for 7 days followed by three thawing methods. ucfDNA, urinary cell-free DNA; DNA yield, pg DNA/mL urine; FWU, frozen whole urine; FUS, frozen urine supernatant; FUSS, frozen urine supernatant with postthawing secondary centrifugation; RT, room temperature; n.s., nonsignificant; *p < 0.05. **p < 0.01. Statistical significance was determined with repeated measures one-way ANOVA (A–F, H–I) and the Friedman test (G).

In the presence of 10 mM EDTA, the US samples maintained similar ucfDNA yields and %cfDNA scores to the baseline after 7 days of RT storage. In contrast, the WU samples presented obviously higher ucfDNA yields but lower %cfDNA scores than the baseline (35.79 ± 0.50 vs 59.13 ± 0.92, S2A and S2B Fig). After being frozen at −20°C for 7 days, the FWU samples thawed at RT and 37°C presented significantly higher ucfDNA yields than did the baseline samples (*p* = 0.0098, *p* = 0.0244, Fig 5A). However, the FWU samples thawed at 4°C presented the lowest ucfDNA yields (with a loss of 61.84%), which were obviously lower than those of the other groups (Fig 5A). Postthawing, all FWU samples presented lower %cfDNA scores than the baseline samples did, regardless of the method used (Fig 5G). The FUS samples presented no significant differences in the ucfDNA yields or %cfDNA scores, regardless of whether the three thawing methods or the baseline methods were compared (Fig 5B and 5H). The DNA electrophoretic bands were also comparable to those at baseline, as shown in Fig 4B. Furthermore, the FWU and FUS samples thawed at RT and 37°C presented comparable DNA profiles to those of the WU and US samples stored for 7 days at RT (S2C and S2D Fig). Despite showing higher %cfDNA scores, the ucfDNA yields of the FUSS samples were lower than those of the baseline samples under the three thawing methods, with a considerable difference observed when the samples were thawed at 4°C (Figs 4B, 5C and 5I).

When thawed at 4°C, only the FUS samples maintained ucfDNA yields comparable to those at baseline (Fig 5D). Upon thawing at RT and 37°C, the FWU samples presented obviously higher ucfDNA yields than the FUS and FUSS samples did (Fig 5E and 5F). While the FUSS samples performed similarly to the FUS samples and baseline samples when thawed at RT and 37°C, a substantial reduction of 80.30% in the ucfDNA yield was observed when the samples were thawed at 4°C (Fig 5D–5F).

In summary, without EDTA, FUS consistently maintained higher ucfDNA yields and %cfDNA scores than the baseline across all thawing methods, while FWU had major losses at 4°C, and FUSS performed consistently poorly. With 10 mM EDTA, FUS was robust to thawing conditions and showed ucfDNA quality comparable to the baseline, FWU samples presented obviously lower %cfDNA scores, and FUSS lost 80.30% ucfDNA yield when thawed at 4°C.

### The impact of storage conditions on ucfDNA quality

**Fig 6:**
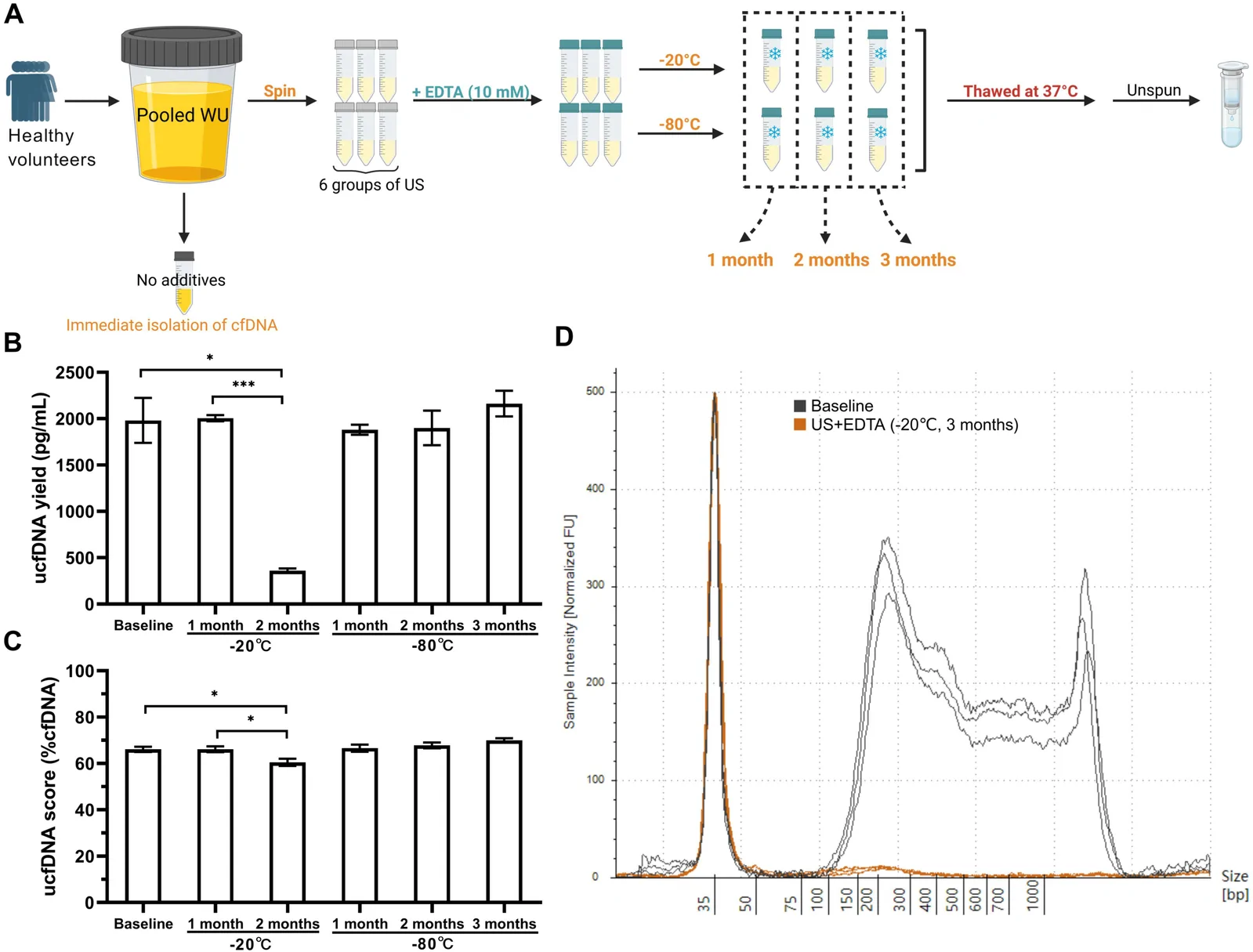
Evaluation of the ucfDNA quality of 10 mM EDTA-treated FUS samples under different storage conditions. (A) Schematic workflow of 10 mM EDTA-treated FUS preprocessing, with triplicates conducted for each group. (B) ucfDNA yields of 10 mM EDTA-treated FUS samples stored at −20°C/-80°C for 1–3 months. (C) The %cfDNA scores of ucfDNA extracted from 10 mM EDTA-treated FUS samples stored at −20°C/-80°C for 1–3 months. (D) Electropherogram profile of ucfDNA extracted from 10 mM EDTA-treated FUS samples stored at −20°C for 3 months. ucfDNA, urinary cell-free DNA; DNA yield, pg DNA/mL urine; WU, whole urine; US, urine supernatant; FUS, frozen urine supernatant; n.s., nonsignificant; *p < 0.05. ***p < 0.001. Statistical significance was determined with a paired *t* tes*t* for samples stored at −20°C and repeated measures one-way ANOVA for samples stored at −80°C (B–C). Fig 6A was generated and permitted by BioRender.

To evaluate the impact of storage conditions on the quality of ucfDNA, the US samples with 10 mM EDTA were stored at −20°C or at −80°C for one to three months (Fig 6A). The yields of ucfDNA from samples stored at −80°C were greater than those from samples stored at −20°C (Fig 6B). There were no significant differences in the ucfDNA yields or %cfDNA scores among the three storage periods or compared with the baseline values when the samples were stored at −80°C (Fig 6B and 6C). However, the yields and %cfDNA scores of ucfDNA from samples stored at −20°C for two months were obviously lower than those of samples stored for one month, and complete ucfDNA degradation occurred after three months of storage (Fig 6B–6D).

### The performance of the optimal preservation protocol in patient urine samples

**Fig 7:**
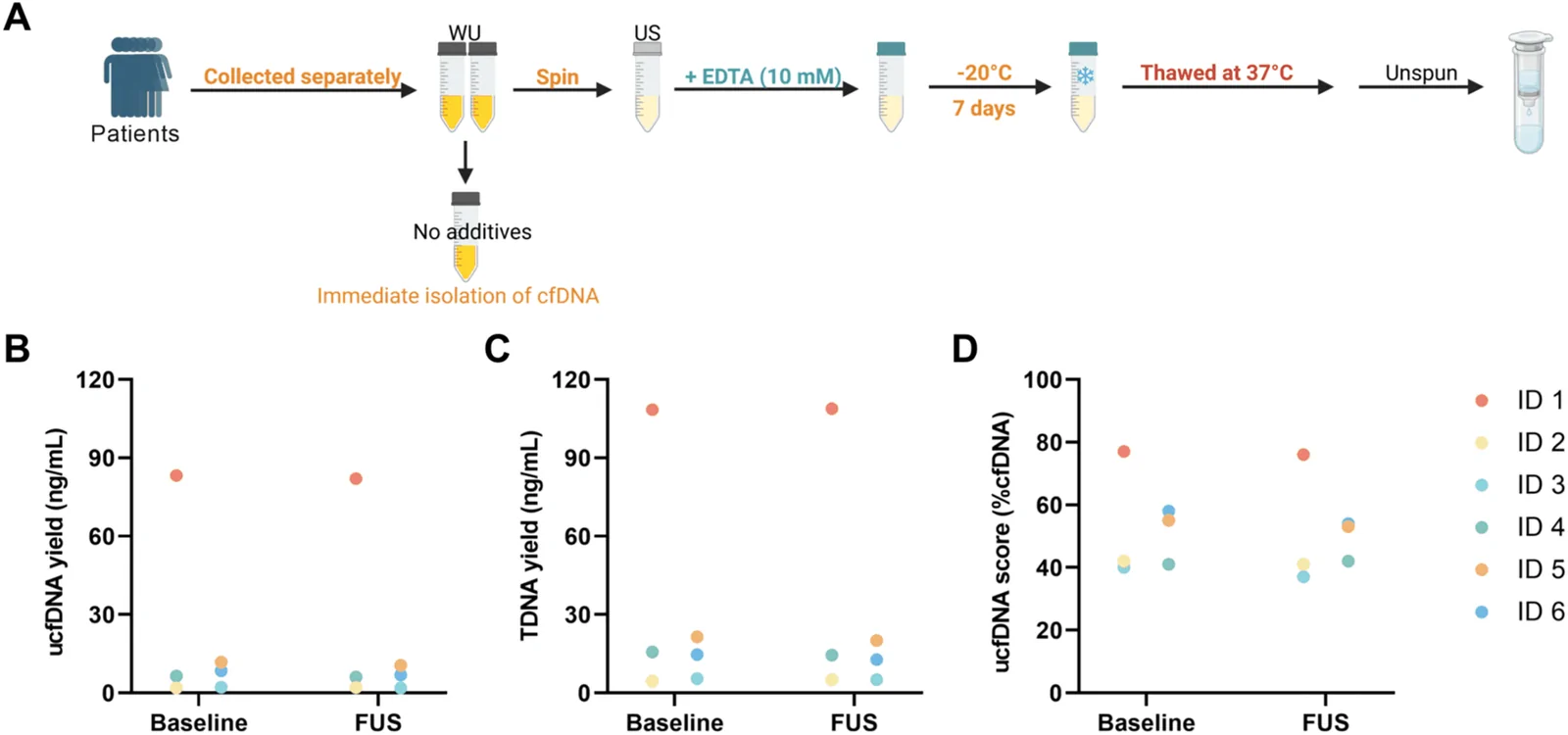
Evaluation of the ucfDNA quality in patient urine samples using an optimal preservation protocol. (A) Schematic workflow of patient urine preprocessing. (B) ucfDNA yields, (C) TDNA yields and (D) %cfDNA scores of ucfDNA extracted from 10 mM EDTA-treated patient US samples that were frozen at −20°C for 7 days and thawed at 37°C. ucfDNA, urinary cell-free DNA; TDNA, total DNA; DNA yield, pg DNA/mL urine; WU, whole urine; US, urine supernatant. Statistical significance was determined with a paired *t* test.

Based on the preceding experiments, the optimal preservation protocol was defined as follows: 10 mM EDTA-treated US, frozen at −20°C for 7 days, and thawed at 37°C prior to extraction. This condition was selected because it maintained baseline-equivalent ucfDNA yields and %cfDNA scores across all tested thawing methods, offering a practical balance between preservation efficacy and operational feasibility. Compared with baseline, ucfDNA quality was preserved at a comparable level when frozen 10 mM EDTA‑treated US (n = 6) was thawed at 37°C (Fig 7B–7D).

## Discussion

Although ucfDNA has been reported as a viable candidate for liquid biopsies, its practical applications may be partly constrained by the absence of well-established standardized preprocessing protocols [10–14]. To identify practical and standard procedures for the preservation of urine samples intended for cfDNA extraction, we investigated the impact of several preanalytical variables on ucfDNA quality.

This study initially assessed the efficiency of EDTA in maintaining ucfDNA quality in both WU and US samples stored at RT. Our findings revealed the varying effects of EDTA on ucfDNA quality in WU and US samples. The rapid degradation of ucfDNA in untreated WU and US after one day of RT storage underscored the necessity of adding preservatives to inhibit nucleases. This finding aligns with previous studies indicating that cfDNA is prone to degradation in unstabilized urine due to the high levels of nucleases such as DNase I and DNase II [1,11,14,15]. Although adding EDTA can preserve ucfDNA for up to 7 days, the substantial yield reduction in the 20 mM and 40 mM groups by day 7 suggests a potential tradeoff between the EDTA concentration and long-term stability.

Notably, the considerable decrease in %cfDNA scores across all EDTA‑treated WU samples resulted from reduced ucfDNA and increased TDNA, suggesting HMW DNA contamination. We hypothesize that while EDTA inhibits nucleases, it cannot prevent cellular lysis. Our data further suggest that higher EDTA concentrations may compromise membrane integrity and promote lysis [27,28]. The mechanistic explanations offered herein, particularly regarding EDTA-induced cellular lysis and the origin of HMW DNA, are inferred from indirect biochemical measurements (TDNA accumulation and %cfDNA decline) and have not been experimentally validated. Direct measurements, such as flow cytometric assessment of cellular integrity or quantification of DNase activity in urine, were not performed in this study. Thus, while our data are consistent with the proposed mechanisms, alternative interpretations cannot be excluded. Future studies incorporating time-course experiments with parallel measurements of cell viability and nuclease activity across a range of EDTA concentrations would be valuable to directly test the mechanistic inferences drawn from our observational data.

In uncentrifuged WU, ongoing cellular lysis releases genomic DNA despite the presence of EDTA, diluting the ucfDNA fraction. Alternative strategies (e.g., rapid freezing, cell preservatives, or protease inhibitors) are needed to mitigate this [29–31]. Moreover, cellular DNA and inherent ucfDNA may also degrade during prolonged RT storage [12,14]. Among commercial preservatives, Streck Urine Preservative and Urinary Analyte Stabilizer have shown promise in limiting cellular lysis and preserving cfDNA integrity in WU [11,15,18,32–34], although further validation is warranted. Compared with the WU samples, the US samples generally presented higher %cfDNA scores. This improvement likely reflects the effectiveness of centrifugation in reducing HMW DNA contamination and enhancing cfDNA purity. Throughout the 7‑day storage period, the 10 mM EDTA‑treated US samples maintained stable ucfDNA yields and %cfDNA scores. In contrast, the 40 mM EDTA group exhibited a marked decline in ucfDNA yields. These observations underscore the importance of selecting appropriate EDTA concentrations for optimal preservation.

The evaluation of thawing methods for frozen urine samples provides critical insights into ucfDNA protection. FUS samples with 10 mM EDTA exhibited robust stability across all thawing methods, likely because centrifugation reduced cellular contamination and EDTA inhibited nuclease activity [15,35,36]. In contrast, FUS without EDTA showed noticeable post-thawing degradation of HMW DNA, which was consistent with concurrent release and degradation in unstabilized urine [14,37,38]. FWU samples with or without EDTA experienced dramatic ucfDNA losses when thawed at 4°C, probably due to cell damage and nuclease release during prolonged ice recrystallization (~16 h) [39–41]. EDTA-treated FWU presented lower %cfDNA scores, reflecting persistent HMW DNA release. This finding was consistent with the results of the EDTA-treated WU samples stored at RT. In addition, comparable electropherogram profiles were observed between RT-stored and rapidly thawed frozen samples, aligning with Kim’s findings [12].

In the absence of EDTA, the FUSS samples yielded unsatisfactory ucfDNA under all thawing methods, particularly when thawed at 4°C. This suggests that postthawing secondary centrifugation adversely impacts the ucfDNA yield, potentially through partial adsorption to the sediment. Even when supplemented with 10 mM EDTA, the FUSS samples presented obviously lower ucfDNA yields when thawed at 4°C, with a loss of 80.30%. This finding is consistent with a previous report by Lee et al., who observed >80% cfDNA loss in EDTA‑treated US samples after 3 months of storage at −70°C, followed by thawing at 4°C and centrifugation [1]. However, EDTA-treated FUSS maintained baseline-equivalent ucfDNA yields when thawed at 37°C and RT. We suppose that rapid thawing protects ucfDNA from degradation, minimizing the negative impact of postthawing secondary centrifugation.

Overall, cellular lysis and DNA degradation occurred concurrently. The addition of EDTA to WU decreased the %cfDNA scores, limiting its utility for WU preservation. In contrast, an appropriate EDTA concentration in US maintained stable ucfDNA yields and %cfDNA scores. Without preservative, frozen urine was unstable during freeze‒thaw. FUS with 10 mM EDTA maintained baseline-equivalent ucfDNA regardless of the thawing method or secondary centrifugation. The decline in ucfDNA quality at −20°C after two months aligns with the findings of Lee et al. [1], whereas −80°C storage with rapid thawing at 37°C preserved stable quality. Rapid thawing minimizes ice-crystal recrystallization and cellular damage. Patient validation confirmed that frozen 10 mM EDTA-treated US thawed at 37°C preserved ucfDNA comparable to baseline, supporting the robustness of this protocol.

The strengths of this study are the investigations of urine preprocessing for both temporary storage (RT or −20°C) and long-term storage, as well as thawing strategies for frozen urine samples. Notably, ucfDNA assessment included not only yield but also purity (%cfDNA score), enabling the selection of workflows that preserve high-quality ucfDNA. The optimized protocol provides a standardized foundation for future biomarker studies. Nevertheless, several limitations should be acknowledged. First, to ensure detectable ucfDNA and sufficient volume for various groupings, we pooled urine samples from different HVs. While pooling minimized inter‑individual variability and enabled experimental control, it may mask individual‑specific factors (e.g., baseline nuclease activity, cellular shedding rates) on EDTA efficacy and degradation dynamics. Moreover, this approach resulted in a limited number of biological replicates per group, which may constrain the statistical power of this study. While the effect sizes reported for most comparisons are large, these estimates may be unstable owing to the limited sample size, and their precision should be evaluated in future replication studies. Accordingly, the present findings should be considered hypothesis-generating and require independent validation in larger cohorts. Second, the baseline ucfDNA level in patient urine samples was much higher than that in HV urine samples and exhibited marked individual variation, leaving it unclear whether the performance of different EDTA concentrations would also differ. Third, the validation using patient samples was limited in size and was restricted to urological tumors. These patient data are preliminary and serve only as supportive evidence that the optimized protocol preserves ucfDNA quality in clinical specimens. Further systematic validation in larger independent cohorts and across other diseases is required before clinical implementation.

## Conclusion

Our findings highlight the critical roles of centrifugation, EDTA concentration, storage conditions, and thawing methods in maintaining ucfDNA quality. Centrifugation prior to storage is the primary determinant of ucfDNA stability. Adding 10 mM EDTA to US is recommended, followed by temporary storage at RT for up to 7 days or freezing at −20°C for less than one month. For long-term storage, ultralow temperatures such as −80°C are necessary. While EDTA effectively prevents rapid ucfDNA degradation, its utility for WU is limited by progressive cellular lysis and HMW DNA contamination. In thawing experiments, FUS samples with 10 mM EDTA exhibited robust stability across all thawing methods, whereas thawing at 4°C severely compromised the FWU and FUSS. Rapid thawing at 37°C or RT can yield good ucfDNA quality. Post-thawing secondary centrifugation of FUS is unnecessary.

## Supporting information

S1 Fig Evaluation of the ucfDNA quality of urine samples without/with EDTA. (A-B) The electropherogram profile of ucfDNA extracted from untreated (0 mM EDTA) (A) WU and (B) US samples stored at RT for 1, 3, and 7 days. (C) The TDNA yields of EDTA-treated (5–40 mM) WU samples stored at RT for 1, 3, and 7 days. ucfDNA, urinary cell-free DNA; TDNA, total DNA; DNA yield, pg DNA/mL urine; WU, whole urine; US, urine supernatant; RT, room temperature.
(TIF)

S2 Fig Evaluation of the ucfDNA quality of 10 mM EDTA-treated urine samples. (A) The ucfDNA yields (B) %cfDNA scores of 10 mM EDTA-treated WU and US samples stored at RT for 7 days. (C) Electropherogram profile of ucfDNA extracted from 10 mM EDTA-treated WU samples stored at RT for 7 days and FWU samples stored at −20°C for 7 days followed by three thawing methods. (D) Electropherogram profile of ucfDNA extracted from 10 mM EDTA-treated US samples stored at RT for 7 days and from FUS samples stored at −20°C for 7 days followed by three thawing methods. ucfDNA, urinary cell-free DNA; DNA yield, pg DNA/mL urine; WU, whole urine; US, urine supernatant; FWU, frozen whole urine; FUS, frozen urine supernatant; RT, room temperature; n.s., nonsignificant; *p < 0.05. ***p < 0.001. ****p < 0.0001. Statistical significance was determined with repeated measures one-way ANOVA.
(TIF)

S1 File Source data and gel images underlying each figure. Raw ucfDNA yields, TDNA yields, %cfDNA scores, and gel images for each replicate.
(XLSX)

## Abbreviations

cfDNA: cell-free DNA
ucfDNA: urinary cell-free DNA
TDNA: Total DNA
HMW DNA: high-molecular-weight DNA
EDTA: ethylenediaminetetraacetic acid
RT: room temperature
WU: whole urine
US: urine supernatant
FUS: frozen urine supernatant
FWU: frozen whole urine
FUSS: FUS with postthawing secondary centrifugation
HVs: healthy volunteers
