# Supplementary material for: Impact of preanalytical variables on urinary cell-free DNA quality: Centrifugation, EDTA concentration, storage and thawing strategies
Source: PLoS One. 2026 Aug 27;21(8):e0356722. doi: 10.1371/journal.pone.0356722 (PMC13521345; doi:10.1371/journal.pone.0356722)
Supplement: Supplementary file 1 [file pone.0356722.s005.doc]

Response Letter

PONE-D-26-22557

Impact of preanalytical variables on urinary cell-free DNA quality: centrifugation, EDTA concentration, storage and thawing strategies

Dear Editor and Reviewers,

We sincerely thank you for your thoughtful and constructive comments on our manuscript. We are very grateful for your positive recognition of our work. We have carefully considered all the points raised and have revised the manuscript accordingly.

Below we provide our point-by-point responses.

***Comments raised by editor***

*1. Please ensure that your manuscript meets PLOS ONE’s style requirements, including those for file naming. The PLOS ONE style templates can be found at https://journals.plos.org/plosone/s/file?id=wjVg/PLOSOne_formatting_sample_main_body.pdf and https://journals.plos.org/plosone/s/file?id=ba62/PLOSOne_formatting_sample_title_authors_affiliations.pdf*

**Re:** We sincerely thank the editor for the kind suggestion on the formatting requirements. We have revised the manuscript in accordance with the guidelines of the PLOS ONE style templates, including the formatting of the abstract, table, references, figure citations, symbols, fonts, and file naming.

*2. Thank you for stating the following in the Acknowledgments Section of your manuscript:*

*“This work was supported by grants from the Hubei Provincial Science and Technology Plan Project (2025CFC012) and the Open Research Fund of Hubei Key Laboratory of Urological Diseases (MNXTJB202414).”*

*We note that you have provided funding information that is not currently declared in your Funding Statement. However, funding information should not appear in the Acknowledgments section or other areas of your manuscript. We will only publish funding information present in the Funding Statement section of the online submission form.*

*Please remove any funding-related text from the manuscript and let us know how you would like to update your Funding Statement. Currently, your Funding Statement reads as follows:*

*“K.Q., Hubei Provincial Science and Technology Plan Project (2025CFC012), Conceptualization, Funding acquisition, Resources,Writing—review and editing. Zhangyan Zhou (Z.Z.), Open Research Fund of Hubei Key Laboratory of Urological Diseases (MNXTJB202414), Conceptualization, Funding acquisition, Project administration, Supervision,Writing—review and editing.”*

*Please include your amended statements within your cover letter; we will change the online submission form on your behalf.*

**Re:** We apologize for the confusion caused by the funding information appearing in the Acknowledgments section. We have removed all funding-related content from the manuscript. We would like to update the Funding Statement in the online submission form as follows:

“This work was supported by grants from the Hubei Provincial Science and Technology Plan Project (grant no. 2025CFC012, recipient K.Q., https://kjt.hubei.gov.cn/kjdt/tzgg/202504/t20250428_5632951.shtml) and the Open Research Fund of Hubei Key Laboratory of Urological Diseases (grant no. MNXTJB202414, recipient Zhangyan Zhou (Z.Z.), https://www.znhospital.com/mnxtjb.html). The funders had no role in study design, data collection and analysis, decision to publish, or preparation of the manuscript.”

We have included this amended statement within our cover letter. We appreciate your assistance in updating the online submission form on our behalf.

*3. Thank you for stating the following financial disclosure:*

*“K.Q., Hubei Provincial Science and Technology Plan Project (2025CFC012), Conceptualization, Funding acquisition, Resources,Writing—review and editing. Zhangyan Zhou (Z.Z.), Open Research Fund of Hubei Key Laboratory of Urological Diseases (MNXTJB202414), Conceptualization, Funding acquisition, Project administration, Supervision,Writing—review and editing.”*

*Please state what role the funders took in the study. If the funders had no role, please state: “The funders had no role in study design, data collection and analysis, decision to publish, or preparation of the manuscript.”*

*If this statement is not correct you must amend it as needed.*

*Please include this amended Role of Funder statement in your cover letter; we will change the online submission form on your behalf.*

**Re:** We apologize for the previous ambiguous description. We confirm that the funders (Hubei Provincial Science and Technology Plan Project and Open Research Fund of Hubei Key Laboratory of Urological Diseases) had no role in study design, data collection and analysis, decision to publish, or preparation of the manuscript.

We have therefore adopted the exact standard statement you provided:

“The funders had no role in study design, data collection and analysis, decision to publish, or preparation of the manuscript.”

This statement has been included in our cover letter as requested. We appreciate your assistance in updating the online submission form accordingly.

*4. In the online submission form you indicate that your data is not available for proprietary reasons and have provided a contact point for accessing this data. Please note that your current contact point is a co-author on this manuscript. According to our Data Policy, the contact point must not be an author on the manuscript and must be an institutional contact, ideally not an individual. Please revise your data statement to a non-author institutional point of contact, such as a data access or ethics committee, and send this to us via return email. Please also include contact information for the third party organization, and please include the full citation of where the data can be found.*

**Re:** We appreciate your reminder regarding the data availability policy. To fully comply with this requirement, we have now uploaded all source data and original gel images as Supplementary Files (S1 File) in the online submission system. Consequently, the data are publicly accessible without restriction, and a third-party contact point is no longer necessary.

We have also revised the Data Availability Statement in the manuscript to reflect this change.

Accordingly, we kindly request that the Data Availability Statement in the online submission form be updated to the following:

“All data generated or analyzed during this study are included in this published article and its supplementary information files.”

We hope this revision satisfactorily resolves the accessibility concern. Thank you for your assistance in updating the form on our behalf.

*5. Your ethics statement should only appear in the Methods section of your manuscript. If your ethics statement is written in any section besides the Methods, please delete it from any other section.*

**Re:** Thank you for your kind reminder. As requested, we have deleted the ethics statement from all other sections of the manuscript, and it now appears solely in the Methods section (lines 117–123).

*6. We note that your paper includes detailed descriptions of individual patients/participants. As per the PLOS ONE policy (http://journals.plos.org/plosone/s/submission-guidelines#loc-human-subjects-research) on papers that include identifying, or potentially identifying, information, the individual(s) or parent(s)/guardian(s) must be informed of the terms of the PLOS open-access (CC-BY) license and provide specific permission for publication of these details under the terms of this license. Please download the Consent Form for Publication in a PLOS Journal (http://journals.plos.org/plosone/s/file?id=8ce6/plos-consent-form-english.pdf). The signed consent form should not be submitted with the manuscript, but should be securely filed in the individual’s case notes. Please amend the methods section and ethics statement of the manuscript to explicitly state that the patient/participant has provided consent for publication: “The individual in this manuscript has given written informed consent (as outlined in PLOS consent form) to publish these case details”.*

**Re:** We greatly appreciate your notification regarding the PLOS ONE policy on papers that include identifying or potentially identifying information. In response, we have downloaded the PLOS Consent Form for Publication, communicated the terms of the PLOS open-access (CC-BY) license to all participants, and obtained explicit written consent for the publication of their case details under this license. The signed consent forms have been securely filed in accordance with the journal’s requirements.

Accordingly, we have revised the Methods section and the Ethics Statement (lines 121–123) to include the required explicit declaration: “All individuals in this study have provided written informed consent (as outlined in the PLOS consent form) to authorize publication of their case details.”

*7. If the reviewer comments include a recommendation to cite specific previously published works, please review and evaluate these publications to determine whether they are relevant and should be cited. There is no requirement to cite these works unless the editor has indicated otherwise.*

**Re:** Thanks a lot for your kind reminder. We have carefully reviewed the reviewer comments and confirm that they do not include any recommendations to cite specific previously published works.

*8. Please review your reference list to ensure that it is complete and correct. If you have cited papers that have been retracted, please include the rationale for doing so in the manuscript text, or remove these references and replace them with relevant current references. Any changes to the reference list should be mentioned in the rebuttal letter that accompanies your revised manuscript. If you need to cite a retracted article, indicate the article’s retracted status in the References list and also include a citation and full reference for the retraction notice.*

**Re:** Thank you for the reminder. All references have been verified against the original sources. We confirm that no cited articles are retracted. We added three references and updated the reference sequence numbers in the revised manuscript, which are listed as follows:

Lines 217–219

25. Fritz CO, Morris PE, Richler JJ. Effect size estimates: current use, calculations, and interpretation. J Exp Psychol Gen. 2012;141(1):2-18. https://doi.org/10.1037/a0024338 PMID: 21823805

26. Fiel Peres F. Effect sizes for nonparametric tests. Biochem Med (Zagreb). 2026; 36:010101. https://doi.org/10.11613/BM.2026.010101 PMID: 41399660

Lines 442–443

28. Vazdar K, Tempra C, Olżyńska A, Biriukov D, Cwiklik L, Vazdar M. Stealthy Player in Lipid Experiments? EDTA Binding to Phosphatidylcholine Membranes Probed by Simulations and Monolayer Experiments. J Phys Chem B. 2023;127(24):5462-5469. https://doi.org/10.1021/acs.jpcb.3c03207. PMID: 37307026

We ensure that the revised reference list is complete and correct.

Furthermore, we have updated the reference format to conform to the journal’s current style, as observed in recently published articles, and have supplemented the citations with DOI and PMID information wherever possible.

***Comments raised by reviewer #1***

*This manuscript systematically evaluates the impact of multiple preanalytical variables (centrifugation, EDTA concentration, storage, and thawing) on urinary cell-free DNA (ucfDNA) quality. The study addresses a highly relevant methodological gap in liquid biopsy research and provides practical recommendations for sample handling. The experimental design is comprehensive and the conclusions are generally supported by the data. However, several issues related to study design, interpretation, and translational applicability should be further strengthened.*

**Re:** We thank the reviewer for the positive assessment of our study’s significance and experimental design. We agree that the aspects of study design, interpretation, and translational applicability deserve further strengthened.

In the revised manuscript, we have

(1) emphasized the limitation of pooling samples and recommended a validation in larger, independent individual cohorts;

(2) clarified that a small patient cohort was designed as a preliminary translatability check rather than a definitive clinical validation;

(3) reframed our mechanistic language from declarative statements to cautious hypotheses, added the explicit discussion of mechanistic interpretations and recommended future time-course validation of cell lysis and nuclease activity;

(4) added effect size estimates for key comparisons, clarified the multiple testing correction that was applied, and discussed the statistical power;

(5) revised minor grammatical inconsistencies, and split overly long sentences to improve readability.

Detailed responses to each point are provided below.

*1) The study primarily relies on pooled urine from healthy volunteers to minimize variability. While this improves experimental control, it substantially limits biological relevance. Pooling masks inter-individual variability, which is critical in real-world applications and findings may not fully reflect variability in patient samples. Suggest to emphasize this limitation more explicitly in the discussion and clarify how pooling may influence EDTA optimization and degradation dynamics. Consider expanding validation in independent individual samples in future work.*

**Re:** Thank you very much for your insightful comments. We fully agree with your assessment regarding the limitations of using pooled urine samples. We would like to clarify the rationale behind this design choice and acknowledge its limitations. We have now explicitly emphasized this limitation in the revised Discussion section (lines 510–513) by adding the following sentences: “While pooling minimized inter‑individual variability and enabled experimental control, it may mask individual‑specific factors (e.g., baseline nuclease activity, cellular shedding rates) on EDTA efficacy and degradation dynamics.” In addition, we have added a future perspective (lines 524–526) recommending validation in larger, independent individual cohorts prior to clinical implementation, as detailed below: “Further systematic validation in larger independent cohorts and across other diseases is required before clinical implementation.”

Regarding the rationale for using pooled samples in this methodological study, we would like to clarify that this was a strategy to establish baseline experimental conditions by minimizing inter-individual variability. This approach enabled the systematic evaluation of preanalytical variables without confounding by biological heterogeneity. Similar pooling strategies have been employed in previous ucfDNA methodological studies. For instance, Nel I et al. pooled urine samples from four male and four female healthy volunteers to ensure identical conditions across aliquots and comparability among experimental groups (Diagnostics, 2023, DOI: 10.3390/diagnostics13243670, which was cited as Ref. 15). Likewise, Jordaens S et al. used female-pooled or male-pooled urine samples to evaluate storage conditions (Cancers, 2023, DOI: 10.3390/cancers15123119, which was cited as Ref. 18). Therefore, we have added the corresponding citations at the first mention of the urine pooling procedure in the Methods section (lines 127–130).

Nevertheless, we fully acknowledge that pooling substantially limits biological relevance, as it masks inter-individual differences in factors such as baseline nuclease activity and cellular shedding rates, which may influence EDTA efficacy and degradation dynamics. Because pooling averages out these individual-specific parameters, the EDTA concentration and storage conditions optimized for pooled samples may not be universally optimal for every individual patient. Consequently, the observed trends (e.g., %cfDNA decline, TDNA accumulation) represent average effects, and individual samples may exhibit more pronounced or milder responses depending on baseline characteristics.

*2) Although the optimized protocol is validated in patient urine, the validation cohort is very small (n=6) and restricted to urological tumors.*

**Re:** Thank you for the comments. We fully agree that the patient validation set is limited in both size (n=6) and disease spectrum (urological tumors). In the revised manuscript, we have clarified that the primary objective of this study was to establish a systematic comparison of preanalytical variables under controlled conditions using pooled healthy volunteer urine. The validation in six patient samples was designed as a proof-of-concept feasibility assessment rather than a definitive clinical validation (lines 103–112).

We acknowledge that the six patients comprised a small series restricted to urological conditions, including renal pelvis cancer, prostate cancer, bladder cancer, and primary hyperaldosteronism (Table 1). While this provides preliminary evidence across distinct pathological entities within the urinary tract, it does not establish generalizability to other tumor types or larger patient populations. In the revised Discussion (lines 521–524), we have emphasized that these patient data are preliminary and serve only as supportive evidence that the optimized protocol preserves ucfDNA quality in clinical specimens. Further systematic validation in larger independent cohorts and across other diseases is required before clinical implementation.

*3) The manuscript provides plausible explanations (e.g., cellular lysis, HMW DNA contamination), but these remain inferred rather than experimentally validated. Mechanistic conclusions (e.g., EDTA-induced membrane destabilization) are not directly tested and no direct measurement of cell lysis or nuclease activity.*

**Re:** Thank you for this insightful comment. We fully agree that our mechanistic interpretations regarding EDTA-induced membrane destabilization, cellular lysis, and HMW DNA accumulation are inferred from indirect evidence (i.e., TDNA accumulation and %cfDNA decline) and were not directly tested in the current study.

We have substantially revised the Discussion to explicitly acknowledge this limitation and to reframe our mechanistic language from declarative statements to cautious hypotheses. Specifically, we have:

(1) Replaced definitive mechanistic language (e.g., “EDTA destabilizes the membrane”) with tentative phrasing (e.g., “We hypothesize that while EDTA inhibits nucleases, it cannot prevent cellular lysis”, “Our data further suggest that higher EDTA concentrations may compromise membrane integrity and promote lysis”).

(2) Included a supporting reference that provides indirect evidence for EDTA-induced membrane destabilization in biological fluids, which we have cited alongside our cautious phrasing (Vazdar K et al., J Phys Chem B, 2023, doi:10.1021/acs.jpcb.3c03207, now cited as Ref. 28 in the revised manuscript).

(3) Added the following explicit discussion of mechanistic interpretations (lines 443–450):

“The mechanistic explanations offered herein, particularly regarding EDTA-induced cellular lysis and the origin of HMW DNA, are inferred from indirect biochemical measurements (TDNA accumulation and %cfDNA decline) and have not been experimentally validated. Direct measurements, such as flow cytometric assessment of cellular integrity or quantification of DNase activity in urine, were not performed in this study. Thus, while our data are consistent with the proposed mechanisms, alternative interpretations cannot be excluded.”

(4) Added a forward-looking recommendation in the Discussion (lines 450–453):

“Future studies incorporating time-course experiments with parallel measurements of cell viability and nuclease activity across a range of EDTA concentrations would be valuable to directly test the mechanistic inferences drawn from our observational data.”

*4) Statistical methods are generally appropriate, but reporting and interpretation could be improved. Include confidence intervals or effect size estimates for key comparisons and clarify whether multiple testing correction was applied. Discuss the limitation of low statistical power more explicitly.*

**Re:** Thank you for the meticulous and constructive suggestions. we have made the following improvements:

(1) Effect size estimates. For all key comparisons, we calculated appropriate effect size measures using IBM SPSS Statistics 20, following established guidelines in the literatures (Fritz CO et al., J Exp Psychol Gen, 2012, DOI: 10.1037/a0024338; Fiel Peres F et al., Biochem Med, 2026, DOI: 10.11613/BM.2026.010101, now cited as Ref. 25 and Ref. 26 in the revised manuscript). We reported partial η² for RM one-way ANOVA, Kendall’s W for Friedman tests, Hedges’ g for paired t-tests, and r for Wilcoxon tests. All comparisons showed large effect sizes, with the exception of one medium effect. The p-values and effect sizes of all key comparisons are summarized in the table below and included in the Supporting Information (S1 File) for straightforward reference. We have also updated the Statistical analysis subsection (lines 217–221).

| **Table: Effect sizes** | | | |
| --- | --- | --- | --- |
| **Fig 1B** | *p* = 0.0472 | Partial η² = 0.8861 | Large effect |
| **Fig 1C** | *p* = 0.1417 | Partial η² = 0.6877 | Large effect |
| **Fig 1D** | *p* = 0.0259 | Kendall’s W = 0.7780 | Large effect |
| **Fig 1E** | *p* = 0.0055 | Partial η² = 0.9621 | Large effect |
| **Fig 1F** | *p* = 0.0037 | Partial η² = 0.9744 | Large effect |
| **Fig 1G** | *p* = 0.0135 | Partial η² = 0.9715 | Large effect |
| **Fig 2B** | *p* = 0.0056 | Kendall’s W = 0.8680 | Large effect |
| **Fig 2C** | *p* = 0.0254 | Partial η² = 0.8845 | Large effect |
| **Fig 2D** | *p* = 0.0195 | Partial η² = 0.9206 | Large effect |
| **Fig 2E** | *p* = 0.2253 | Partial η² = 0.5357 | Large effect |
| **Fig 2F** | *p* = 0.0796 | Partial η² = 0.8017 | Large effect |
| **Fig 2G** | *p* = 0.0022 | Partial η² = 0.9688 | Large effect |
| **Fig 3B** | *p* = 0.0001 | Partial η² = 0.9922 | Large effect |
| **Fig 3C** | *p* = 0.0728 | Partial η² = 0.7486 | Large effect |
| **Fig 3D** | *p* = 0.0008 | Partial η² = 0.9898 | Large effect |
| **Fig 3E** | *p* = 0.0002 | Partial η² = 0.9916 | Large effect |
| **Fig 3F** | *p* = 0.0008 | Partial η² = 0.9875 | Large effect |
| **Fig 3G** | *p* = 0.0141 | Partial η² = 0.9456 | Large effect |
| **Fig 3H** | *p* = 0.0017 | Kendall’s W = 1.0000 | Large effect |
| **Fig 3I** | *p* = 0.0052 | Partial η² = 0.9701 | Large effect |
| **Fig 3J** | *p* = 0.0894 | Partial η² = 0.8286 | Large effect |
| **Fig 5A** | *p* = 0.0002 | Partial η² = 0.9861 | Large effect |
| **Fig 5B** | *p* = 0.4625 | Partial η² = 0.2919 | Large effect |
| **Fig 5C** | *p* = 0.0056 | Partial η² = 0.9771 | Large effect |
| **Fig 5D** | *p* = 0.0094 | Partial η² = 0.9740 | Large effect |
| **Fig 5E** | *p* = 0.0027 | Partial η² = 0.9719 | Large effect |
| **Fig 5F** | *p* = 0.0064 | Partial η² = 0.9680 | Large effect |
| **Fig 5G** | *p* = 0.0538 | Kendall’s W = 0.7780 | Large effect |
| **Fig 5H** | *p* = 0.0823 | Partial η² = 0.7964 | Large effect |
| **Fig 5I** | *p* = 0.0017 | Kendall’s W = 1.0000 | Large effect |
| **Fig 6B** | *p* = 0.0064 | Partial η² = 0.9669 | Large effect |
| **Fig 6C** | *p* = 0.0129 | Partial η² = 0.8887 | Large effect |
| **Fig 7B** | *p* = 0.2188 | r = 0.5765 | Large effect |
| **Fig 7C** | *p* = 0.0625 | r = 0.3935 | Medium effect |
| **Fig 7D** | *p* = 0.0671 | Hedges’ g = 1.8978 | Large effect |
| **S2A Fig** | *p* = 0.0017 | Partial η² = 0.9966 | Large effect |
| **S2B Fig** | *p* < 0.0001 | Partial η² = 0.9998 | Large effect |
| **Interpretation thresholds:** | | | |
| Partial η² (small ≥ 0.01, medium ≥ 0.06, large ≥ 0.14) | | | |
| Kendall’s W (small ≥ 0.1, medium ≥ 0.3, large ≥ 0.5) | | | |
| Hedges’ g (small ≥ 0.2, medium ≥ 0.5, large ≥ 0.8) | | | |
| r (small ≥ 0.1, medium ≥ 0.3, large ≥ 0.5) | | | |

(2) Multiple testing correction. We have explicitly clarified in the Statistical analysis subsection (lines 212–215) that, following a significant overall result (RM one-way ANOVA or Friedman test), post hoc pairwise comparisons were performed with appropriate correction: Tukey’s or Dunnett’s test for parametric repeated-measures ANOVA, and Dunn’s test for non-parametric Friedman post hoc comparisons. Thus, the reported pairwise p-values reflect adjustment for multiple comparisons.

(3) Low statistical power. We fully agree that the limited number of biological replicates substantially reduces statistical power, which may increase the risk of Type II errors (i.e., failing to detect true differences) and renders some non-significant comparisons inconclusive rather than truly negative. Additionally, although the observed effect sizes are generally large, these estimates may be imprecise due to the small sample size and should be interpreted with caution. To address this explicitly, we have added the following limitation to the Discussion section (lines 513–518): “Moreover, this approach resulted in a limited number of biological replicates per group, which may constrain the statistical power of this study. While the effect sizes reported for most comparisons are large, these estimates may be unstable owing to the limited sample size, and their precision should be evaluated in future replication studies. Accordingly, the present findings should be considered hypothesis-generating and require independent validation in larger cohorts.”

*5) Overall language is clear and professional. Minor grammatical inconsistencies and long sentences are present. Perform minor language polishing to improve readability.*

**Re:** Thank you very much for the positive and encouraging assessment of our language quality. We have taken your suggestion and have carefully proofread the entire manuscript to address minor grammatical inconsistencies and overlong sentences. All textual changes are highlighted in the revised manuscript for your easy reference. Here are two representative examples.

Example 1: “minor grammatical inconsistencies”

Before revision (lines 61–64):

“This issue may arise because the numerous microorganisms present in urine can easily cause sample deterioration, high nuclease activity makes ucfDNA more prone to degradation, and ucfDNA is highly fragmented [2,15].”

After revision (lines 59–62):

“This issue may arise because: (1) numerous microorganisms in urine can easily cause sample deterioration; (2) high nuclease activity makes ucfDNA prone to degradation; and (3) ucfDNA is highly fragmented [2,15].”

Example 2: “overlong sentences”

Before revision (lines 66–72):

“Preservatives such as ethylenediaminetetraacetic acid (EDTA) without or with adjusted pH, the “AlloU” buffer established by researchers, and proprietary commercial stabilizers (e.g., Streck Urine Preservative, Urine Conservation Medium, Urine Conservation Buffer, Urinary Analyte Stabilizer) have been added to either the unprocessed whole urine (WU) or the urine supernatant (US) after centrifugation to investigate their protective effects on ucfDNA [1,5,11–12,15–18].”

After revision (lines 64–70):

“The protective effects of various preservatives on ucfDNA have been investigated. These include ethylenediaminetetraacetic acid (EDTA) (with or without pH adjustment), the “AlloU” buffer, and commercial stabilizers such as Streck Urine Preservative, Urine Conservation Medium, Urine Conservation Buffer, and Urinary Analyte Stabilizer [1,5,11–12,15–18]. They were added to either unprocessed whole urine (WU) or urine supernatant (US) after centrifugation.”

Additionally, we have engaged the Research Square Language Quality service to perform a professional language check as following:

***Comments raised by reviewer #2***

*This manuscript studies the effect of preanalytical factors (EDTA concentration, centrifugation, storage temperature and time, and thawing conditions) on ucfDNA yield and %cfDNA score of ucfDNA using pooled urine from healthy volunteers to optimize the urine preprocessing methods, and validates the optimal protocol using patient urine samples. The experimental design is appropriate for optimizing and standardizing ucfDNA processing workflows, and the validation using patient urine samples provides additional support for the applicability of the optimized procedure. This sample handling standardization study is within the scope of PLOS One.*

**Re:** We greatly appreciate the reviewer for the positive assessment of our experimental design and for recognizing that our study falls within the scope of PLOS ONE. We have carefully addressed all the minor comments raised and revised the manuscript accordingly. In the revised manuscript, we have

(1) added summary statements of main results;

(2) provided an explicit optimized protocol summary for patient sample validation;

(3) refined data description of the untreated (0 mM EDTA) US samples at Day 1;

(4) converted the abstract to a non-structured, single-paragraph format and streamlined the abstract;

(5) included a supplementary data file (source data and original gel images).

Below, we provide our point-by-point responses.

*Here are some minor comments I have.*

*1)The result section describes detailed findings of individual experiments, but would benefit from brief summary statements highlighting the key conclusions from each optimization step. This would improve readability and help readers follow the overall rationale.*

**Re:** Thank you for your suggestion. We agree that summary statements would enhance the readability and logical flow of the Results section. In the revised manuscript, we have added a concluding summary sentence at the end of each subsection (lines 241–244, 276–278, and 364–369) to clearly state the key takeaway from each optimization step, helping readers follow the overall rationale without getting lost in experimental details.

For example, we have added the following summary for the evaluation of ucfDNA preservation in EDTA-treated WU (lines 241–244):

“Collectively, these results revealed a key limitation of EDTA in WU. Although it preserved ucfDNA yield, it failed to prevent progressive HMW DNA contamination, as indicated by the declining %cfDNA scores.”

All newly added summaries are highlighted in the revised manuscript for easy identification.

*2) Before presenting the patient sample validation, the authors should explicitly summarize the optimized preservation protocol identified from the preceding experiments. Currently, the selected condition can be inferred from the Methods section but is not clearly stated in the Results section, making the transition to validation less straightforward.*

**Re:** Thank you for your suggestion. We agree that explicitly stating the optimized protocol prior to the patient validation section would improve the logical transition. In the revised manuscript, we have added an explicit summary of the optimized protocol immediately before the patient validation subsection (lines 408–412). The added text reads:

“Based on the preceding experiments, the optimal preservation protocol was defined as follows: 10 mM EDTA-treated US, frozen at -20°C for 7 days, and thawed at 37°C prior to extraction. This condition was selected because it maintained baseline-equivalent ucfDNA yields and %cfDNA scores across all tested thawing methods, offering a practical balance between preservation efficacy and operational feasibility.”

This statement now provides a clear transition from the optimization experiments to the patient validation. The added text is highlighted in the revised manuscript (lines 408–412).

*3) In Evaluation of ucfDNA Preservation in EDTA-treated US in the result section, it is stated that “Complete ucfDNA degradation also occurred in untreated (0 mM EDTA) US samples after 1 day of RT storage (S1B Fig).” This statement seems stronger than supported by the data as low-level 50–700 bp DNA signals remain detectable for Day 1 (0 mM EDTA) triplicates in the electropherogram. A description such as “substantial degradation” or “marked reduction” may more accurately reflect the results.*

**Re:** We thank the reviewer for this careful observation. Upon re-examination of the electropherogram data, we agree that trace levels of 50–700 bp DNA signals remain detectable in the untreated (0 mM EDTA) US samples at Day 1, and the term “complete degradation” is indeed too strong. We have revised the description to more accurately reflect the data.

Abstract (lines 29–31): untreated whole urine (WU) and urine supernatant (US) both exhibited substantial ucfDNA degradation within 1 day at RT.

Results (lines 261–262): substantial ucfDNA degradation occurred in untreated (0 mM EDTA) US samples after 1 day of RT storage (S1B Fig).

*4) The abstract is currently presented in a structured format (Background/Results/Conclusions). Since the journal guidelines (https://journals.plos.org/plosone/s/submission-guidelines.) do not appear to explicitly require this format, the authors may consider confirming that it aligns with the journal**’s preferred style. Additionally, the abstract could be streamlined to emphasize the major findings and significance of the study.*

**Re:** We appreciate the reviewer’s attention to this formatting detail. Following a comprehensive review of the PLOS ONE submission guidelines and the articles recently published in this journal, we have converted the abstract to a non-structured, single-paragraph format. The revised abstract has been streamlined to emphasizes the key findings and practical recommendations (lines 24–46). The revised abstract now reads:

“Urinary cell-free DNA (ucfDNA) holds promise for liquid biopsies, yet standardized preprocessing protocols remain to be fully established. Using pooled urine from healthy volunteers, we compared the effects of EDTA concentration (0–40 mM), centrifugation (0–2 spins), storage temperature and duration (RT, -20°C, -80°C), and thawing conditions (4°C, RT, 37°C) on ucfDNA yield and purity (%cfDNA score). The optimal protocol was subsequently validated using patient urine. Untreated whole urine (WU) and urine supernatant (US) both exhibited substantial ucfDNA degradation within 1 day at RT. For EDTA-treated WU, %cfDNA scores declined markedly owing to high-molecular-weight DNA contamination, and 20–40 mM EDTA compromised yields. In contrast, 10 mM EDTA-treated US optimally preserved both ucfDNA yields and %cfDNA scores for 7 days at RT, whereas 40 mM EDTA impaired yields. Frozen US showed thawing-method-independent stability, whereas frozen WU thawed at 4°C lost >60% of ucfDNA yields. Post-thawing secondary centrifugation further reduced ucfDNA recovery. For long-term storage, 10 mM EDTA-treated US maintained baseline-equivalent ucfDNA quality at -80°C for 3 months, but ucfDNA degraded after 2 months at -20°C. Patient urine validation confirmed that frozen 10 mM EDTA-treated US frozen at -20°C and thawed at 37°C preserved ucfDNA quality comparable to baseline. This study offers practical guidelines and standardized preprocessing protocols for optimizing ucfDNA preservation. For temporary storage of urine intended for ucfDNA preservation, adding EDTA to WU is not advised; instead, we recommend supplementing 10 mM EDTA to US and storing at RT ≤ 7 days or at -20°C ≤ one month. Long-term storage requires temperatures below -80°C. Rapid thawing can yield good ucfDNA quality.”

*5) The manuscript presents many quantitative results as bar graphs with summary statistics (mean ± error bars), but the underlying individual data points do not appear to be provided. According to the journal’s data availability policy, the authors should consider providing the source data used to generate each figure in the supplementary materials.*

**Re:** We thank the reviewer for this important reminder. PLOS ONE encourages authors to make “the numerical data underlying each of the main and supplementary figures available to readers, either as supplementary data files or as depositions in an online repository.”

In response, we have prepared a comprehensive supplementary data file (S1 File) that contains all individual data points and gel images underlying each figure in the main manuscript. This file includes raw ucfDNA yields, TDNA yields, %cfDNA scores, effect sizes and gel images for each replicate in every experimental group. The source data file has been uploaded as Supporting Information with the designation S1 File.

Accordingly, we have updated the Data Availability Statement in the manuscript to:

“All data generated or analyzed during this study are included in this published article and its supplementary information files (S1 File).”

*Overall, the study provides insights regarding ucfDNA sample handling and preservation. While the current evaluation focuses on ucfDNA yield and %cfDNA score rather than specific molecular biomarkers, these parameters are appropriate for assessing processing efficiency and sample quality.*

**Re:** We are grateful for the reviewer’s positive assessment of our study’s contribution to ucfDNA sample handling and preservation, and we appreciate the recognition that ucfDNA yield and %cfDNA score are appropriate parameters for evaluating processing efficiency and sample quality.

We fully agree that the present evaluation focuses on quality metrics rather than disease-specific molecular biomarkers. This was a staged design. We established robust preprocessing standards first, because reliable downstream biomarker analysis depends on that baseline. We fully concur with the reviewer that the ultimate translational value depends on how these processing parameters affect specific molecular biomarkers, such as mutation detection or methylation patterns.

We have now explicitly acknowledged this in the revised Discussion (lines 507–508), stating that the optimized protocol provides a standardized foundation for future biomarker studies. Furthermore, we are currently planning follow-up studies to validate the impact of these preprocessing conditions on clinically relevant molecular biomarkers.

Thank you again for this constructive suggestion, which has helped clarify an important direction for our follow-up investigations.
