# Supplementary material for: Impact of preanalytical variables on urinary cell-free DNA quality: Centrifugation, EDTA concentration, storage and thawing strategies
Source: PLoS One. 2026 Aug 27;21(8):e0356722. doi: 10.1371/journal.pone.0356722 (PMC13521345; doi:10.1371/journal.pone.0356722)
Supplement: Peer review history

# Peer review: Decision Letter 0

**DOI:** 10.1371/journal.pone.0356722.r001

**Authors:** Zheng Yuan

18 Jun 2026

Dear Dr. Zhang,

Thank you for submitting your manuscript to PLOS ONE. After careful consideration, we feel that it has merit but does not fully meet PLOS ONE’s publication criteria as it currently stands. Therefore, we invite you to submit a revised version of the manuscript that addresses the points raised during the review process.

Please submit your revised manuscript by Jul 18 2026 11:59PM. If you will need more time than this to complete your revisions, please reply to this message or contact the journal office at plosone@plos.org. When you're ready to submit your revision, log on to https://www.editorialmanager.com/pone/ and select the 'Submissions Needing Revision' folder to locate your manuscript file.

- A letter that responds to each point raised by the academic editor and reviewer(s). You should upload this letter as a separate file labeled 'Response to Reviewers'.
- A marked-up copy of your manuscript that highlights changes made to the original version. You should upload this as a separate file labeled 'Revised Manuscript with Track Changes'.
- An unmarked version of your revised paper without tracked changes. You should upload this as a separate file labeled 'Manuscript'.

If you would like to make changes to your financial disclosure, please include your updated statement in your cover letter. Guidelines for resubmitting your figure files are available below the reviewer comments at the end of this letter.

If applicable, we recommend that you deposit your laboratory protocols in protocols.io to enhance the reproducibility of your results. Protocols.io assigns your protocol its own identifier (DOI) so that it can be cited independently in the future. For instructions see: https://journals.plos.org/plosone/s/submission-guidelines#loc-laboratory-protocols. Additionally, PLOS ONE offers an option for publishing peer-reviewed Lab Protocol articles, which describe protocols hosted on protocols.io. Read more information on sharing protocols at https://plos.org/protocols?utm_medium=editorial-email&utm_source=authorletters&utm_campaign=protocols.

As the corresponding author, your ORCID iD is verified in the submission system and will appear in the published article. PLOS supports the use of ORCID, and we encourage all coauthors to register for an ORCID iD and use it as well. Please encourage your coauthors to verify their ORCID iD within the submission system before final acceptance, as unverified ORCID iDs will not appear in the published article. *Only*  the individual author can complete the verification step; PLOS staff *cannot*  verify ORCID iDs on behalf of authors.

We look forward to receiving your revised manuscript.

Kind regards,

Zheng Yuan

Academic Editor

PLOS One

Journal Requirements:

When submitting your revision, we need you to address these additional requirements.

1. Please ensure that your manuscript meets PLOS ONE's style requirements, including those for file naming. The PLOS ONE style templates can be found at

https://journals.plos.org/plosone/s/file?id=wjVg/PLOSOne_formatting_sample_main_body.pdf and

https://journals.plos.org/plosone/s/file?id=ba62/PLOSOne_formatting_sample_title_authors_affiliations.pdf

2. Thank you for stating the following in the Acknowledgments Section of your manuscript:

“This work was supported by grants from the Hubei Provincial Science and Technology Plan Project (2025CFC012) and the Open Research Fund of Hubei Key Laboratory of Urological Diseases (MNXTJB202414).”

We note that you have provided funding information that is not currently declared in your Funding Statement. However, funding information should not appear in the Acknowledgments section or other areas of your manuscript. We will only publish funding information present in the Funding Statement section of the online submission form.

Please remove any funding-related text from the manuscript and let us know how you would like to update your Funding Statement. Currently, your Funding Statement reads as follows:

“K.Q., Hubei Provincial Science and Technology Plan Project (2025CFC012), Conceptualization, Funding acquisition, Resources,Writing—review and editing. Zhangyan Zhou (Z.Z.), Open Research Fund of Hubei Key Laboratory of Urological Diseases (MNXTJB202414), Conceptualization, Funding acquisition, Project administration, Supervision,Writing—review and editing.”

Please include your amended statements within your cover letter; we will change the online submission form on your behalf.

3. Thank you for stating the following financial disclosure:

“K.Q., Hubei Provincial Science and Technology Plan Project (2025CFC012), Conceptualization, Funding acquisition, Resources,Writing—review and editing. Zhangyan Zhou (Z.Z.), Open Research Fund of Hubei Key Laboratory of Urological Diseases (MNXTJB202414), Conceptualization, Funding acquisition, Project administration, Supervision,Writing—review and editing.”

Please state what role the funders took in the study.  If the funders had no role, please state: "The funders had no role in study design, data collection and analysis, decision to publish, or preparation of the manuscript."

If this statement is not correct you must amend it as needed.

Please include this amended Role of Funder statement in your cover letter; we will change the online submission form on your behalf.

4. In the online submission form you indicate that your data is not available for proprietary reasons and have provided a contact point for accessing this data. Please note that your current contact point is a co-author on this manuscript. According to our Data Policy, the contact point must not be an author on the manuscript and must be an institutional contact, ideally not an individual. Please revise your data statement to a non-author institutional point of contact, such as a data access or ethics committee, and send this to us via return email. Please also include contact information for the third party organization, and please include the full citation of where the data can be found.

5. Your ethics statement should only appear in the Methods section of your manuscript. If your ethics statement is written in any section besides the Methods, please delete it from any other section.

6. We note that your paper includes detailed descriptions of individual patients/participants. As per the PLOS ONE policy (http://journals.plos.org/plosone/s/submission-guidelines#loc-human-subjects-research) on papers that include identifying, or potentially identifying, information, the individual(s) or parent(s)/guardian(s) must be informed of the terms of the PLOS open-access (CC-BY) license and provide specific permission for publication of these details under the terms of this license. Please download the Consent Form for Publication in a PLOS Journal (http://journals.plos.org/plosone/s/file?id=8ce6/plos-consent-form-english.pdf). The signed consent form should not be submitted with the manuscript, but should be securely filed in the individual's case notes. Please amend the methods section and ethics statement of the manuscript to explicitly state that the patient/participant has provided consent for publication: “The individual in this manuscript has given written informed consent (as outlined in PLOS consent form) to publish these case details

7. If the reviewer comments include a recommendation to cite specific previously published works, please review and evaluate these publications to determine whether they are relevant and should be cited. There is no requirement to cite these works unless the editor has indicated otherwise.

8. Please review your reference list to ensure that it is complete and correct. If you have cited papers that have been retracted, please include the rationale for doing so in the manuscript text, or remove these references and replace them with relevant current references. Any changes to the reference list should be mentioned in the rebuttal letter that accompanies your revised manuscript. If you need to cite a retracted article, indicate the article’s retracted status in the References list and also include a citation and full reference for the retraction notice.

[Note: HTML markup is below. Please do not edit.]

Reviewers' comments:

Reviewer's Responses to Questions

**Comments to the Author**

1. Is the manuscript technically sound, and do the data support the conclusions?

Reviewer #1: Yes

Reviewer #2: Yes

**********

2. Has the statistical analysis been performed appropriately and rigorously?

Reviewer #1: Yes

Reviewer #2: Yes

**********

3. Have the authors made all data underlying the findings in their manuscript fully available?

The PLOS Data policy

Reviewer #1: Yes

Reviewer #2: No

**********

4. Is the manuscript presented in an intelligible fashion and written in standard English?

Reviewer #1: Yes

Reviewer #2: Yes

**********

Reviewer #1: This manuscript systematically evaluates the impact of multiple preanalytical variables (centrifugation, EDTA concentration, storage, and thawing) on urinary cell-free DNA (ucfDNA) quality. The study addresses a highly relevant methodological gap in liquid biopsy research and provides practical recommendations for sample handling. The experimental design is comprehensive and the conclusions are generally supported by the data. However, several issues related to study design, interpretation, and translational applicability should be further strengthened.

1) The study primarily relies on pooled urine from healthy volunteers to minimize variability. While this improves experimental control, it substantially limits biological relevance. Pooling masks inter-individual variability, which is critical in real-world applications and findings may not fully reflect variability in patient samples. Suggest to emphasize this limitation more explicitly in the discussion and clarify how pooling may influence EDTA optimization and degradation dynamics. Consider expanding validation in independent individual samples in future work.

2) Although the optimized protocol is validated in patient urine, the validation cohort is very small (n=6) and restricted to urological tumors.

3) The manuscript provides plausible explanations (e.g., cellular lysis, HMW DNA contamination), but these remain inferred rather than experimentally validated.Mechanistic conclusions (e.g., EDTA-induced membrane destabilization) are not directly tested and no direct measurement of cell lysis or nuclease activity.

4) Statistical methods are generally appropriate, but reporting and interpretation could be improved. Include confidence intervals or effect size estimates for key comparisons and clarify whether multiple testing correction was applied. Discuss the limitation of low statistical power more explicitly.

5) Overall language is clear and professional. Minor grammatical inconsistencies and long sentences are present. Perform minor language polishing to improve readability.

Reviewer #2: This manuscript studies the effect of preanalytical factors (EDTA concentration, centrifugation, storage temperature and time, and thawing conditions) on ucfDNA yield and %cfDNA score of ucfDNA using pooled urine from healthy volunteers to optimize the urine preprocessing methods, and validates the optimal protocol using patient urine samples. The experimental design is appropriate for optimizing and standardizing ucfDNA processing workflows, and the validation using patient urine samples provides additional support for the applicability of the optimized procedure. This sample handling standardization study is within the scope of PLOS One.

Here are some minor comments I have.

1. The result section describes detailed findings of individual experiments, but would benefit from brief summary statements highlighting the key conclusions from each optimization step. This would improve readability and help readers follow the overall rationale.

2. Before presenting the patient sample validation, the authors should explicitly summarize the optimized preservation protocol identified from the preceding experiments. Currently, the selected condition can be inferred from the Methods section but is not clearly stated in the Results section, making the transition to validation less straightforward.

3. In Evaluation of ucfDNA Preservation in EDTA-treated US in the result section, it is stated that “Complete ucfDNA degradation also occurred in untreated (0 mM EDTA) US samples after 1 day of RT storage (S1B Fig).” This statement seems stronger than supported by the data as low-level 50-700 bp DNA signals remain detectable for Day 1 (0 mM EDTA) triplicates in the electropherogram. A description such as "substantial degradation" or "marked reduction" may more accurately reflect the results.

4. The abstract is currently presented in a structured format (Background/Results/Conclusions). Since the journal guidelines (https://journals.plos.org/plosone/s/submission-guidelines.) do not appear to explicitly require this format, the authors may consider confirming that it aligns with the journal's preferred style. Additionally, the abstract could be streamlined to emphasize the major findings and significance of the study.

5. The manuscript presents many quantitative results as bar graphs with summary statistics (mean ± error bars), but the underlying individual data points do not appear to be provided. According to the journal's data availability policy, the authors should consider providing the source data used to generate each figure in the supplementary materials.

Overall, the study provides insights regarding ucfDNA sample handling and preservation. While the current evaluation focuses on ucfDNA yield and %cfDNA score rather than specific molecular biomarkers, these parameters are appropriate for assessing processing efficiency and sample quality.

**********

what does this mean?). If published, this will include your full peer review and any attached files.

If you choose “no”, your identity will remain anonymous but your review may still be made public.

**Do you want your identity to be public for this peer review?**  For information about this choice, including consent withdrawal, please see our Privacy Policy

Reviewer #1: **Yes:** Panpan GU

Reviewer #2: No

**********

[NOTE: If reviewer comments were submitted as an attachment file, they will be attached to this email and accessible via the submission site. Please log into your account, locate the manuscript record, and check for the action link "View Attachments". If this link does not appear, there are no attachment files.]

To ensure your figures meet our technical requirements, please review our figure guidelines: https://journals.plos.org/plosone/s/figures

You may also use PLOS’s free figure tool, NAAS, to help you prepare publication quality figures: https://journals.plos.org/plosone/s/figures#loc-tools-for-figure-preparation.

NAAS will assess whether your figures meet our technical requirements by comparing each figure against our figure specifications.

# Author comment: Author response to Decision Letter 1

**DOI:** 10.1371/journal.pone.0356722.r002

20 Jul 2026

Response Letter

PONE-D-26-22557

Impact of preanalytical variables on urinary cell-free DNA quality: centrifugation, EDTA concentration, storage and thawing strategies

Dear Editor and Reviewers,

We sincerely thank you for your thoughtful and constructive comments on our manuscript. We are very grateful for your positive recognition of our work. We have carefully considered all the points raised and have revised the manuscript accordingly.

Below we provide our point-by-point responses.

Comments raised by editor

1. Please ensure that your manuscript meets PLOS ONE’s style requirements, including those for file naming. The PLOS ONE style templates can be found at https://journals.plos.org/plosone/s/file?id=wjVg/PLOSOne_formatting_sample_main_body.pdf and https://journals.plos.org/plosone/s/file?id=ba62/PLOSOne_formatting_sample_title_authors_affiliations.pdf

Re: We sincerely thank the editor for the kind suggestion on the formatting requirements. We have revised the manuscript in accordance with the guidelines of the PLOS ONE style templates, including the formatting of the abstract, table, references, figure citations, symbols, fonts, and file naming.

2. Thank you for stating the following in the Acknowledgments Section of your manuscript:

“This work was supported by grants from the Hubei Provincial Science and Technology Plan Project (2025CFC012) and the Open Research Fund of Hubei Key Laboratory of Urological Diseases (MNXTJB202414).”

We note that you have provided funding information that is not currently declared in your Funding Statement. However, funding information should not appear in the Acknowledgments section or other areas of your manuscript. We will only publish funding information present in the Funding Statement section of the online submission form.

Please remove any funding-related text from the manuscript and let us know how you would like to update your Funding Statement. Currently, your Funding Statement reads as follows:

“K.Q., Hubei Provincial Science and Technology Plan Project (2025CFC012), Conceptualization, Funding acquisition, Resources,Writing—review and editing. Zhangyan Zhou (Z.Z.), Open Research Fund of Hubei Key Laboratory of Urological Diseases (MNXTJB202414), Conceptualization, Funding acquisition, Project administration, Supervision,Writing—review and editing.”

Please include your amended statements within your cover letter; we will change the online submission form on your behalf.

Re: We apologize for the confusion caused by the funding information appearing in the Acknowledgments section. We have removed all funding-related content from the manuscript. We would like to update the Funding Statement in the online submission form as follows:

“This work was supported by grants from the Hubei Provincial Science and Technology Plan Project (grant no. 2025CFC012, recipient K.Q., https://kjt.hubei.gov.cn/kjdt/tzgg/202504/t20250428_5632951.shtml) and the Open Research Fund of Hubei Key Laboratory of Urological Diseases (grant no. MNXTJB202414, recipient Zhangyan Zhou (Z.Z.), https://www.znhospital.com/mnxtjb.html). The funders had no role in study design, data collection and analysis, decision to publish, or preparation of the manuscript.”

We have included this amended statement within our cover letter. We appreciate your assistance in updating the online submission form on our behalf.

3. Thank you for stating the following financial disclosure:

“K.Q., Hubei Provincial Science and Technology Plan Project (2025CFC012), Conceptualization, Funding acquisition, Resources,Writing—review and editing. Zhangyan Zhou (Z.Z.), Open Research Fund of Hubei Key Laboratory of Urological Diseases (MNXTJB202414), Conceptualization, Funding acquisition, Project administration, Supervision,Writing—review and editing.”

Please state what role the funders took in the study. If the funders had no role, please state: “The funders had no role in study design, data collection and analysis, decision to publish, or preparation of the manuscript.”

If this statement is not correct you must amend it as needed.

Please include this amended Role of Funder statement in your cover letter; we will change the online submission form on your behalf.

Re: We apologize for the previous ambiguous description. We confirm that the funders (Hubei Provincial Science and Technology Plan Project and Open Research Fund of Hubei Key Laboratory of Urological Diseases) had no role in study design, data collection and analysis, decision to publish, or preparation of the manuscript.

We have therefore adopted the exact standard statement you provided:

“The funders had no role in study design, data collection and analysis, decision to publish, or preparation of the manuscript.”

This statement has been included in our cover letter as requested. We appreciate your assistance in updating the online submission form accordingly.

4. In the online submission form you indicate that your data is not available for proprietary reasons and have provided a contact point for accessing this data. Please note that your current contact point is a co-author on this manuscript. According to our Data Policy, the contact point must not be an author on the manuscript and must be an institutional contact, ideally not an individual. Please revise your data statement to a non-author institutional point of contact, such as a data access or ethics committee, and send this to us via return email. Please also include contact information for the third party organization, and please include the full citation of where the data can be found.

Re: We appreciate your reminder regarding the data availability policy. To fully comply with this requirement, we have now uploaded all source data and original gel images as Supplementary Files (S1 File) in the online submission system. Consequently, the data are publicly accessible without restriction, and a third-party contact point is no longer necessary.

We have also revised the Data Availability Statement in the manuscript to reflect this change.

Accordingly, we kindly request that the Data Availability Statement in the online submission form be updated to the following:

“All data generated or analyzed during this study are included in this published article and its supplementary information files.”

We hope this revision satisfactorily resolves the accessibility concern. Thank you for your assistance in updating the form on our behalf.

5. Your ethics statement should only appear in the Methods section of your manuscript. If your ethics statement is written in any section besides the Methods, please delete it from any other section.

Re: Thank you for your kind reminder. As requested, we have deleted the ethics statement from all other sections of the manuscript, and it now appears solely in the Methods section (lines 117–123).

6. We note that your paper includes detailed descriptions of individual patients/participants. As per the PLOS ONE policy (http://journals.plos.org/plosone/s/submission-guidelines#loc-human-subjects-research) on papers that include identifying, or potentially identifying, information, the individual(s) or parent(s)/guardian(s) must be informed of the terms of the PLOS open-access (CC-BY) license and provide specific permission for publication of these details under the terms of this license. Please download the Consent Form for Publication in a PLOS Journal (http://journals.plos.org/plosone/s/file?id=8ce6/plos-consent-form-english.pdf). The signed consent form should not be submitted with the manuscript, but should be securely filed in the individual’s case notes. Please amend the methods section and ethics statement of the manuscript to explicitly state that the patient/participant has provided consent for publication: “The individual in this manuscript has given written informed consent (as outlined in PLOS consent form) to publish these case details”.

Re: We greatly appreciate your notification regarding the PLOS ONE policy on papers that include identifying or potentially identifying information. In response, we have downloaded the PLOS Consent Form for Publication, communicated the terms of the PLOS open-access (CC-BY) license to all participants, and obtained explicit written consent for the publication of their case details under this license. The signed consent forms have been securely filed in accordance with the journal’s requirements.

Accordingly, we have revised the Methods section and the Ethics Statement (lines 121–123) to include the required explicit declaration: “All individuals in this study have provided written informed consent (as outlined in the PLOS consent form) to authorize publication of their case details.”

7. If the reviewer comments include a recommendation to cite specific previously published works, please review and evaluate these publications to determine whether they are relevant and should be cited. There is no requirement to cite these works unless the editor has indicated otherwise.

Re: Thanks a lot for your kind reminder. We have carefully reviewed the reviewer comments and confirm that they do not include any recommendations to cite specific previously published works.

8. Please review your reference list to ensure that it is complete and correct. If you have cited papers that have been retracted, please include the rationale for doing so in the manuscript text, or remove these references and replace them with relevant current references. Any changes to the reference list should be mentioned in the rebuttal letter that accompanies your revised manuscript. If you need to cite a retracted article, indicate the article’s retracted status in the References list and also include a citation and full reference for the retraction notice.

Re: Thank you for the reminder. All references have been verified against the original sources. We confirm that no cited articles are retracted. We added three references and updated the reference sequence numbers in the revised manuscript, which are listed as follows:

Lines 217–219

25. Fritz CO, Morris PE, Richler JJ. Effect size estimates: current use, calculations, and interpretation. J Exp Psychol Gen. 2012;141(1):2-18. https://doi.org/10.1037/a0024338 PMID: 21823805

26. Fiel Peres F. Effect sizes for nonparametric tests. Biochem Med (Zagreb). 2026; 36:010101. https://doi.org/10.11613/BM.2026.010101 PMID: 41399660

Lines 442–443

28. Vazdar K, Tempra C, Olżyńska A, Biriukov D, Cwiklik L, Vazdar M. Stealthy Player in Lipid Experiments? EDTA Binding to Phosphatidylcholine Membranes Probed by Simulations and Monolayer Experiments. J Phys Chem B. 2023;127(24):5462-5469. https://doi.org/10.1021/acs.jpcb.3c03207. PMID: 37307026

We ensure that the revised reference list is complete and correct.

Furthermore, we have updated the reference format to conform to the journal’s current style, as observed in recently published articles, and have supplemented the citations with DOI and PMID information wherever possible.

Comments raised by reviewer #1

This manuscript systematically evaluates the impact of multiple preanalytical variables (centrifugation, EDTA concentration, storage, and thawing) on urinary cell-free DNA (ucfDNA) quality. The study addresses a highly relevant methodological gap in liquid biopsy research and provides practical recommendations for sample handling. The experimental design is comprehensive and the conclusions are generally supported by the data. However, several issues related to study design, interpretation, and translational applicability should be further strengthened.

Re: We thank the reviewer for the positive assessment of our study’s significance and experimental design. We agree that the aspects of study design, interpretation, and translational applicability deserve further strengthened.

In the revised manuscript, we have

(1) emphasized the limitation of pooling samples and recommended a validation in larger, independent individual cohorts;

(2) clarified that a small patient cohort was designed as a preliminary translatability check rather than a definitive clinical validation;

(3) reframed our mechanistic language from declarative statements to cautious hypotheses, added the explicit discussion of mechanistic interpretations and recommended future time-course validation of cell lysis and nuclease activity;

(4) added effect size estimates for key comparisons, clarified the multiple testing correction that was applied, and discussed the statistical power;

(5) revised minor grammatical inconsistencies, and split overly long sentences to improve readability.

Detailed responses to each point are provided below.

1) The study primarily relies on pooled urine from healthy volunteers to minimize variability. While this improves experimental control, it substantially limits biological relevance. Pooling masks inter-individual variability, which is critical in real-world applications and findings may not fully reflect variability in patient samples. Suggest to emphasize this limitation more explicitly in the discussion and clarify how pooling may influence EDTA optimization and degradation dynamics. Consider expanding validation in independent individual samples in future work.

Re: Thank you very much for your insightful comments. We fully agree with your assessment regarding the limitations of using pooled urine samples. We would like to clarify the rationale behind this design choice and acknowledge its limitations. We have now explicitly emphasized this limitation in the revised Discussion section (lines 510–513) by adding the following sentences: “While pooling minimized inter‑individual variability and enabled experimental control, it may mask individual‑specific factors (e.g., baseline nuclease activity, cellular shedding rates) on EDTA efficacy and degradation dynamics.” In addition, we have added a future perspective (lines 524–526) recommending validation in larger, independent individual cohorts prior to clinical implementation, as detailed below: “Further systematic validation in larger independent cohorts and across other diseases is required before clinical implementation.”

Regarding the rationale for using pooled samples in this methodological study, we would like to clarify that this was a strategy to establish baseline experimental conditions by minimizing inter-individual variability. This approach enabled the systematic evaluation of preanalytical variables without confounding by biological heterogeneity. Similar pooling strategies have been employed in previous ucfDNA methodological studies. For instance, Nel I et al. pooled urine samples from four male and four female healthy volunteers to ensure identical conditions across aliquots and comparability among experimental groups (Diagnostics, 2023, DOI: 10.3390/diagnostics13243670, which was cited as Ref. 15). Likewise, Jordaens S et al. used female-pooled or male-pooled urine samples to evaluate storage conditions (Cancers, 2023, DOI: 10.3390/cancers15123119, which was cited as Ref. 18). Therefore, we have added the corresponding citations at the first mention of the urine pooling procedure in the Methods section (lines 127–130).

Nevertheless, we fully acknowledge that pooling substantially limits biological relevance, as it masks inter-individual differences in factors such as baseline nuclease activity and cellular shedding rates, which may influence EDTA efficacy and degradation dynamics. Because pooling averages out these individual-specific parameters, the EDTA concentration and storage conditions optimized for pooled samples may not be universally optimal for every individual patient. Consequently, the observed trends (e.g., %cfDNA decline, TDNA accumulation) represent average effects, and individual samples may exhibit more pronounced or milder responses depending on baseline characteristics.

2) Although the optimized protocol is validated in patient urine, the validation cohort is very small (n=6) and restricted to urological tumors.

Re: Thank you for the comments. We fully agree that the patient validation set is limited in both size (n=6) and disease spectrum (urological tumors). In the revised manuscript, we have clarified that the primary objective of this study was to establish a systematic comparison of preanalytical va

Attachment Submitted filename: Response to Reviewers.doc

# Peer review: Decision Letter 1

**DOI:** 10.1371/journal.pone.0356722.r003

**Authors:** Zheng Yuan

6 Aug 2026

Impact of preanalytical variables on urinary cell-free DNA quality: centrifugation, EDTA concentration, storage and thawing strategies

PONE-D-26-22557R1

Dear Dr. Zhang,

We’re pleased to inform you that your manuscript has been judged scientifically suitable for publication and will be formally accepted for publication once it meets all outstanding technical requirements.

Within one week, you’ll receive an e-mail detailing the required amendments. When these have been addressed, you’ll receive a formal acceptance letter and your manuscript will be scheduled for publication.

An invoice will be generated when your article is formally accepted. Please note, if your institution has a publishing partnership with PLOS and your article meets the relevant criteria, all or part of your publication costs will be covered. Please make sure your user information is up-to-date by logging into Editorial Manager at Editorial Manager® and clicking the ‘Update My Information' link at the top of the page. For questions related to billing, please contact billing support.

If your institution or institutions have a press office, please notify them about your upcoming paper to help maximize its impact. If they’ll be preparing press materials, please inform our press team as soon as possible -- no later than 48 hours after receiving the formal acceptance. Your manuscript will remain under strict press embargo until 2 pm Eastern Time on the date of publication. For more information, please contact onepress@plos.org.

Kind regards,

Zheng Yuan

Academic Editor

PLOS One

Additional Editor Comments (optional):

Reviewers' comments:

Reviewer's Responses to Questions

**Comments to the Author**

Reviewer #1: All comments have been addressed

Reviewer #2: (No Response)

**********

2. Is the manuscript technically sound, and do the data support the conclusions?

Reviewer #1: Yes

Reviewer #2: Yes

**********

3. Has the statistical analysis been performed appropriately and rigorously?

Reviewer #1: Yes

Reviewer #2: Yes

**********

4. Have the authors made all data underlying the findings in their manuscript fully available?

The PLOS Data policy

Reviewer #1: Yes

Reviewer #2: Yes

**********

5. Is the manuscript presented in an intelligible fashion and written in standard English?

Reviewer #1: Yes

Reviewer #2: Yes

**********

Reviewer #1: My comments/questions are well addressed by the author. I have no specific questions/comments now. The article is now fine for publishing

Reviewer #2: The authors have carefully addressed my previous comments, and the manuscript has been improved. The revised version is clear and well organized overall. I have only a few minor language and clarity suggestions that may further improve the readability of the manuscript.

1. In the abstract, the sentence “Patient urine validation confirmed that frozen 10 mM EDTA-treated US frozen at -20°C and thawed at 37°C preserved ucfDNA quality comparable to baseline.” (line 39-41) contains redundant use of "frozen" and is somewhat difficult to read. Please consider revising the sentence for clarity.

2. The sentence “To address these issues, the primary objective of this study was to establish…” (line 103) reads somewhat awkwardly. Please consider revising the sentence for improved clarity and flow.

Overall, I am satisfied with the revised manuscript and believe the remaining minor comments can be readily addressed. I have no further substantive concerns.

**********

what does this mean?). If published, this will include your full peer review and any attached files.

If you choose “no”, your identity will remain anonymous but your review may still be made public.

**Do you want your identity to be public for this peer review?**  For information about this choice, including consent withdrawal, please see our Privacy Policy

Reviewer #1: **Yes:** GU PANPAN

Reviewer #2: No

**********

# Editor report: Acceptance letter

**DOI:** 10.1371/journal.pone.0356722.r004

**Authors:** Zheng Yuan

PONE-D-26-22557R1

PLOS One

Dear Dr. Zhang,

I'm pleased to inform you that your manuscript has been deemed suitable for publication in PLOS One. Congratulations! Your manuscript is now being handed over to our production team.

At this stage, our production department will prepare your paper for publication. This includes ensuring the following:

* All references, tables, and figures are properly cited

* All relevant supporting information is included in the manuscript submission,

* There are no issues that prevent the paper from being properly typeset

You will receive further instructions from the production team, including instructions on how to review your proof when it is ready. Please keep in mind that we are working through a large volume of accepted articles, so please give us a few days to review your paper and let you know the next and final steps.

Lastly, if your institution or institutions have a press office, please let them know about your upcoming paper now to help maximize its impact. If they'll be preparing press materials, please inform our press team within the next 48 hours. Your manuscript will remain under strict press embargo until 2 pm Eastern Time on the date of publication. For more information, please contact onepress@plos.org.

You will receive an invoice from PLOS for your publication fee after your manuscript has reached the completed accept phase. If you receive an email requesting payment before acceptance or for any other service, this may be a phishing scheme. Learn how to identify phishing emails and protect your accounts at https://explore.plos.org/phishing.

If we can help with anything else, please email us at customercare@plos.org.

Thank you for submitting your work to PLOS One and supporting open access.

Kind regards,

PLOS ONE Editorial Office Staff

on behalf of

Dr. Zheng Yuan

Academic Editor

PLOS One
